# Secretion and Signaling Activities of Lipoprotein-Associated Hedgehog and Non-Sterol-Modified Hedgehog in Flies and Mammals

**DOI:** 10.1371/journal.pbio.1001505

**Published:** 2013-03-12

**Authors:** Wilhelm Palm, Marta M. Swierczynska, Veena Kumari, Monika Ehrhart-Bornstein, Stefan R. Bornstein, Suzanne Eaton

**Affiliations:** 1 Max-Planck Institute of Molecular Cell Biology and Genetics, Dresden, Germany; 2 Department of Internal Medicine III, Carl Gustav Carus Medical School, Technical University of Dresden, Dresden, Germany; MRC-National Institute for Medical Research, United Kingdom

## Abstract

Hedgehog (Hh) proteins control animal development and tissue homeostasis. They activate gene expression by regulating processing, stability, and activation of Gli/Cubitus interruptus (Ci) transcription factors. Hh proteins are secreted and spread through tissue, despite becoming covalently linked to sterol during processing. Multiple mechanisms have been proposed to release Hh proteins in distinct forms; in *Drosophila*, lipoproteins facilitate long-range Hh mobilization but also contain lipids that repress the pathway. Here, we show that mammalian lipoproteins have conserved roles in Sonic Hedgehog (Shh) release and pathway repression. We demonstrate that lipoprotein-associated forms of Hh and Shh specifically block lipoprotein-mediated pathway inhibition. We also identify a second conserved release form that is not sterol-modified and can be released independently of lipoproteins (Hh-N*/Shh-N*). Lipoprotein-associated Hh/Shh and Hh-N*/Shh-N* have complementary and synergistic functions. In *Drosophila* wing imaginal discs, lipoprotein-associated Hh increases the amount of full-length Ci, but is insufficient for target gene activation. However, small amounts of non-sterol-modified Hh synergize with lipoprotein-associated Hh to fully activate the pathway and allow target gene expression. The existence of Hh secretion forms with distinct signaling activities suggests a novel mechanism for generating a diversity of Hh responses.

## Abstract 2

We identify two distinct Hh secretion forms with complementary signaling activities produced in both mammals and flies: one sterol-modified and lipoprotein-associated, and another lacking sterol modification.

## Author Summary

Hedgehog (Hh) proteins are conserved secreted signaling molecules that regulate embryonic development and adult tissue homeostasis. Ectopic Hh signaling promotes tumorigenesis, and secretion of mammalian Sonic Hedgehog (Shh) by many tumors supports their growth and survival. As Hh proteins are covalently modified by sterol and palmitate, specific mechanisms are required to release them from cell membranes. Here, we show that different fly and mammalian cell types, including Shh-dependent cancer cells, release lipid-modified Hh/Shh on lipoproteins—the major lipid carriers in circulation. Perturbed lipoprotein metabolism is a hallmark of metabolic syndrome, which is associated with many tissue pathologies and an elevated cancer risk. We show that *Drosophila* and mammalian lipoproteins act positively to mobilize lipid-modified Hh proteins, but also contain lipids that repress the pathway when Hh/Shh is absent. Association of Hh/Shh with lipoproteins neutralizes their inhibitory effect in both organisms. We also find that many cells release a second form of Hh/Shh independently of lipoproteins that lacks sterol modification. This form cannot overcome lipoprotein-mediated repression but further activates the pathway in the presence of lipoprotein-associated Hh/Shh. The existence of multiple release forms with different activities suggests novel conserved mechanisms for generating diverse responses to Hh ligands. Our data also suggest that perturbed Hh signaling could contribute to human pathologies associated with lipoprotein dysfunction.

## Introduction

Hedgehog (Hh) family proteins are secreted signaling molecules that control animal development and tissue homeostasis [1],[2]. They are produced as precursors that are processed through autocatalytic cleavage and sterol modification to generate the active N-terminal signaling domain [3],[4], which can be further modified by palmitoylation [5]. Both lipid modifications influence Hh signaling, but their precise function remains unclear [6].

Release of dually lipid-modified proteins from cell membranes must involve specific secretion mechanisms. Work in different systems has suggested several mechanisms to release Hh from cellular membranes with its lipid moieties intact, including exovesicles [7], multimers [8]–[11], and lipoproteins [12]. Although the *Drosophila* lipoprotein Lipophorin (Lpp) promotes long-range Hh spreading in the wing imaginal disc, Hh can still signal over shorter distances in Lpp RNAi animals. Thus, wing discs may also secrete Hh by Lpp-independent mechanisms.

In addition to mobilizing Hh, Lpp contains lipids that repress the pathway in the absence of Hh [13]. When Hh is absent, signaling by the 7-pass transmembrane protein Smoothened (Smo) is catalytically repressed by the Hh receptor, Patched (Ptc) [14]. Ptc is thought to repress Smo by regulating access to lipophilic ligands that alter Smo trafficking and activity [15]–[17]. Ptc can mobilize Lpp sterols from endosomes, suggesting that Ptc may modulate Smo activity, in part, by regulating access to inhibitory Lpp lipids [13].

Smo signals by regulating processing of Gli family transcription factors [Cubitus Interruptus (Ci) in *Drosophila*] [18],[19]. When Smo is inactive, these are processed to transcriptional repressors [20]. Active Smo stabilizes full-length Gli/Ci proteins, but to induce target gene transcription, Gli/Ci activation requires additional Smo-dependent events that are not well understood [21],[22]. Lpp specifically promotes degradation of full-length Ci_155_, but does not influence its activation; upon Lpp RNAi, Ci_155_ accumulates, but this does not suffice for target gene transcription [13].

Thus far, the function of lipoproteins in the Hh pathway has only been investigated in *Drosophila*. In this study, we demonstrate conserved release mechanisms for *Drosophila* Hh and human Shh. Both proteins can be secreted in two forms by many different cell types—one is lipoprotein-associated and sterol-modified, the other lacks sterol modification and can be released independently of lipoproteins. Lipoprotein-associated forms of Hh and Shh are also observed in systemic circulation of fruit flies and humans. The lipoprotein-associated forms of Hh/Shh can alleviate the repressive activity of lipoproteins on Hh signaling. Additional signals by non-sterol-modified Hh proteins further activate the pathway to effect target gene expression.

## Results

### Human and *Drosophila* Hh Proteins Are Secreted in Both Lipoprotein-Associated and Lipoprotein-Free Forms

**Figure 1:**
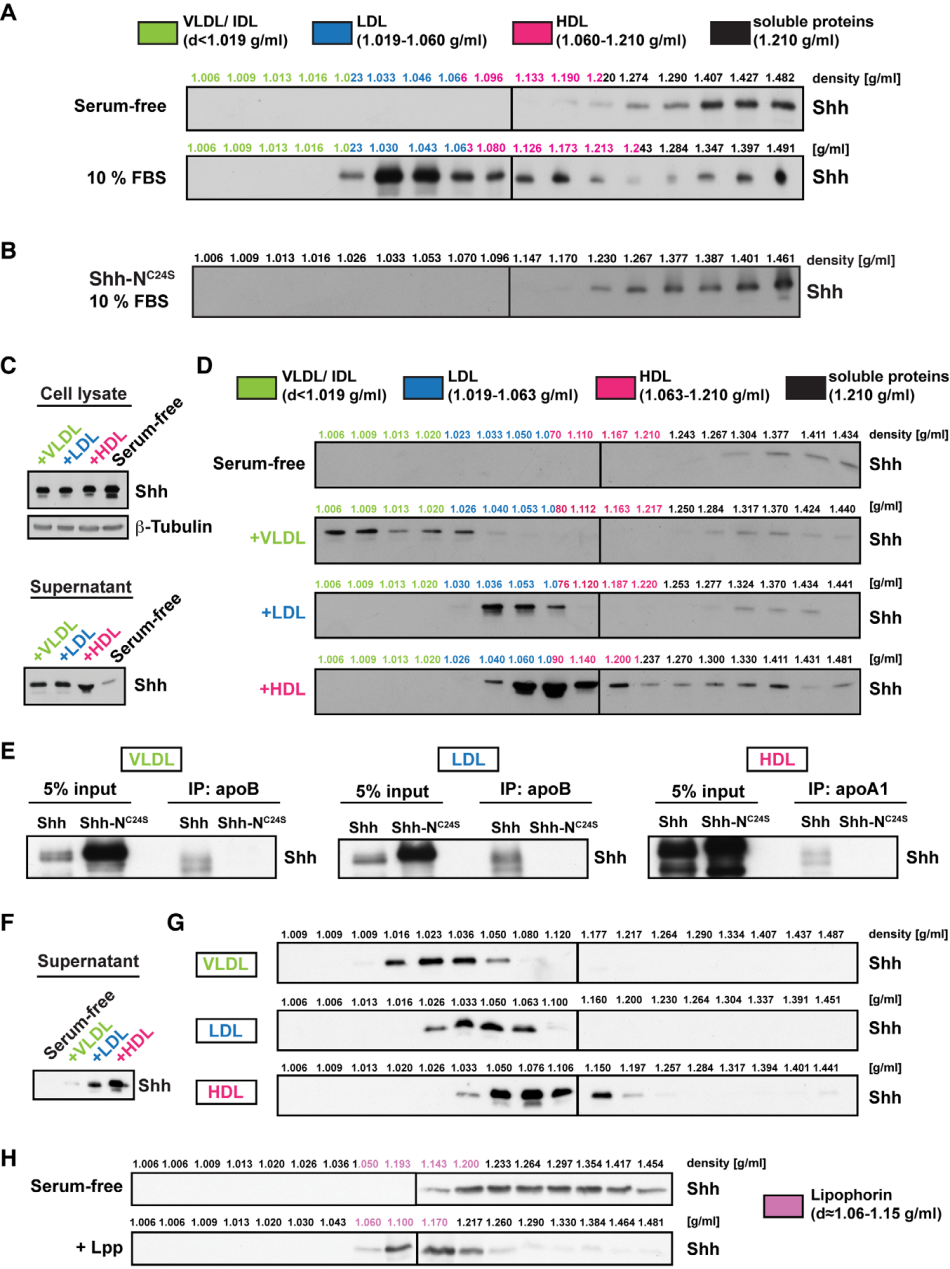
Shh is secreted in lipoprotein-associated and lipoprotein-free forms. (A) Density of human Shh secreted by HeLa cells in the absence or presence of fetal bovine serum (FBS), analyzed by Optiprep density gradient centrifugation, and Western blotting (WB). HeLa cells transfected with Shh were grown in serum-free medium or in the presence of 10% FBS, and equal volumes of supernatants analyzed. Colors indicate fractions corresponding to bovine Very Low-, Low-, and High-Density Lipoproteins (VLDL, LDL, and HDL) [68]. (B) Density of non-lipid-modified Shh-N^C24S^, analyzed by Optiprep density gradient centrifugation and WB. Supernatants were derived from HeLa cells transfected with Shh-N^C24S^ and grown in the presence of FBS. (C) Shh levels in cell lysates and supernatants derived from HeLa cells transfected with Shh, grown in serum-free medium supplemented with individual human lipoprotein classes. Equal protein amounts (cell lysates) or volumes (supernatants) were analyzed. (D) Density of Shh in HeLa cell supernatants shown in (C), analyzed by Optiprep density gradient centrifugation and WB. Colors indicate fractions corresponding to human VLDL, LDL, and HDL [43]. (E) Co-Immunoprecipitation (Co-IP) of secreted Shh with different lipoprotein classes, analyzed by WB. Supernatants were derived from HeLa cells transfected with Shh or Shh-N^C24S^, grown in serum-free medium supplemented with individual human lipoproteins classes. (F) Shh levels in supernatants derived from MIA PaCa-2 cells grown in serum-free medium supplemented with individual human lipoprotein classes. Equal volumes were used for WB. (G) Density of Shh in MIA PaCa-2 cell supernatants shown in (F), analyzed by Optiprep density gradient centrifugation and WB. (H) Density of Shh in supernatants from Shh-expressing HeLa cells grown in serum-free medium supplemented with hemolymph from *Drosophila* larvae, analyzed by Optiprep density gradient centrifugation and WB. Purple indicates fractions corresponding to *Drosophila* Lpp.

To investigate the secretion forms of mammalian Shh, we first transfected HeLa cells with Shh, cultured them in serum-free medium, and assayed Shh in supernatants after 48 h. Isopycnic density centrifugation showed that all detectable Shh is present in high-density fractions (Figure 1A). Thus, HeLa cells do not secrete Shh on lipoproteins, or other low-density particles like exovesicles, in the absence of extrinsically added factors. We then asked whether HeLa cells might release Shh on exogenously added lipoproteins by providing fetal bovine serum to Shh-transfected HeLa cells and assaying Shh in density-fractionated supernatants after 48 h. These supernatants still contain high-density Shh; however, an additional, more abundant pool of Shh is found in fractions corresponding in density to lipoproteins (Figure 1A). This increase in the level of secreted Shh is not caused by increased Shh production (Figure S1A), raising the possibility that serum lipoproteins facilitate the release of Shh by HeLa cells. Transfecting HeLa cells with different Shh lipid modification mutants shows that lipid modification is essential for formation of the low-density secretion form; Shh-N^C24S^, which lacks both cholesteryl and palmitoyl modification, is present exclusively in high-density fractions (Figures 1B and S1B). Thus, HeLa cells can secrete Shh in two forms, one of which depends on extrinsic factors and lipid modification.

To ask whether serum increased Shh release or promoted its stability in supernatants, we followed the accumulation of Shh in supernatants over time. Low-density Shh is already detected in supernatants 1 h after addition of fresh serum-containing medium to Shh-transfected HeLa cells (Figure S1C). In contrast, high-density Shh is only detected after 4 h. This suggests that serum directly increases secretion of a low-density form of Shh.

To test directly whether low-density Shh corresponded to a lipoprotein-associated form, we added specific human lipoprotein classes to Shh-transfected HeLa cells—very low-density lipoprotein (VLDL), low-density lipoprotein (LDL), and high-density lipoprotein (HDL). Western blotting cell supernatants showed that VLDL, LDL, or HDL increases levels of Shh in supernatants without affecting Shh levels in cell lysates (Figure 1C). In density gradients, most Shh fractionates at a density characteristic of the added lipoprotein (Figure 1D). To ask whether Shh bound directly to lipoproteins, we immunoprecipitated lipoproteins from supernatants containing either lipid-modified Shh or non-lipid-modified Shh-N^C24S^. Shh, but not Shh-N^C24S^, is efficiently co-immunoprecipitated by antibodies to proteins that scaffold the major human lipoproteins (Figures 1E and S1D). Thus, Shh associates directly with all major human lipoprotein classes through its lipid moieties.

Secretion of Hh proteins by a variety of cancer types promotes tumor growth [23]. To investigate the secretion forms of Shh endogenously produced by cancer cells, we analyzed supernatants of the pancreatic cancer cell line MIA PaCa-2 grown in serum-free medium supplemented with VLDL, LDL, or HDL [24]. MIA PaCa-2 cells produce much lower levels of Shh than transfected HeLa cells (Figure S2A). However, similar to what we observed in HeLa cells, addition of individual lipoprotein classes strongly increases the amount of Shh present in supernatants (Figure 1F). Furthermore, density gradients show that secreted Shh is present in fractions characteristic of the added lipoprotein (Figures 1G and S2B). Very minor amounts of Shh can also sometimes be detected in high-density fractions. Thus, MIA PaCa-2 cells, which endogenously produce Shh, can release it on the different human lipoprotein classes.

Shh is efficiently mobilized by three different lipoproteins of widely different size and density, scaffolded by two distinct apolipoproteins, suggesting broad specificity in the mechanisms that promote Shh/lipoprotein association. To ask whether these mechanisms might operate across phyla, we investigated whether *Drosophila* Lpp could act as a carrier for human Shh. Indeed, adding *Drosophila* hemolymph to serum-free medium induces HeLa cells to release Shh that co-fractionates with Lpp (Figure 1H). Conversely, Hh-expressing *Drosophila* S2 cells are capable of secreted Hh in a low-density form when grown in the presence of either Lpp or mammalian lipoproteins provided in fetal bovine serum (Figure S2C,D). These data suggest that conserved biophysical properties of lipoproteins, rather than specific protein–protein interactions, promote their association with Hh proteins. Interestingly, *Drosophila* S2 cells also produce a high-density form of Hh similar to the high-density Shh observed in HeLa cell supernatants.

### *Drosophila* Hh Can Be Secreted Into the Hemolymph in Both Lipoprotein-Associated and Lipoprotein-Free Forms

**Figure 2:**
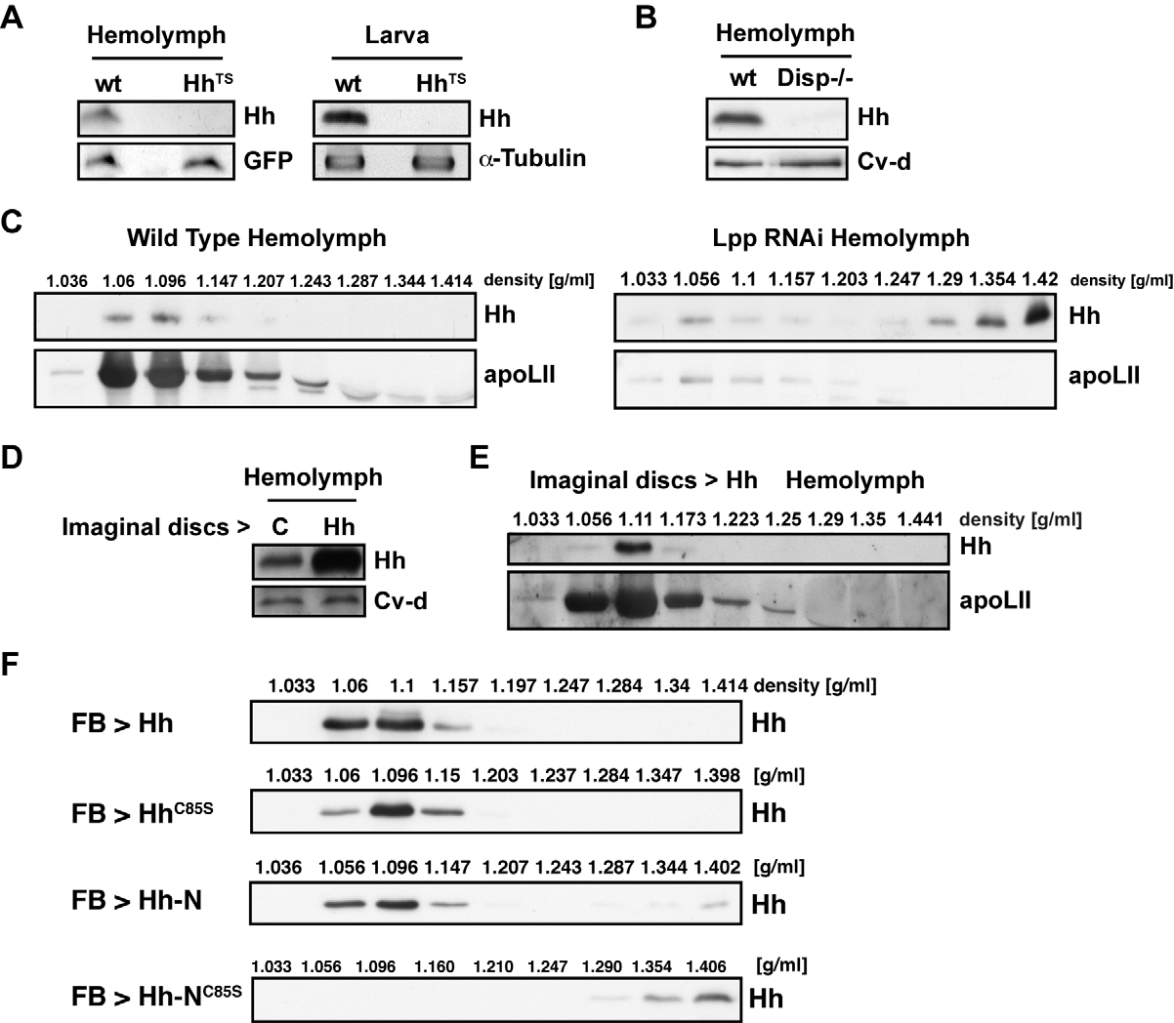
*Drosophila* Hh is secreted in Lpp-associated and Lpp-free forms. (A) Hh levels in hemolymph and whole extracts of wild-type and *hh^TS^* larvae at restrictive temperature, analyzed by WB. Hemolymph loading control is a secreted GFP expressed from the tubulin promoter. (B) Hemolymph Hh levels in wild-type and *disp* mutant larvae, analyzed by WB. Loading control is Cv-d. (C) Density of Hh in hemolymph of wild-type and Lpp RNAi larvae, analyzed by Optiprep density gradient centrifugation and WB. Equal amounts of hemolymph (normalized by protein) were analyzed. (D) Hemolymph Hh levels in larvae overexpressing Hh in imaginal discs with *en105*-GAL4, analyzed by WB. Loading control is Cv-d. (E) Density of hemolymph Hh in larvae overexpressing Hh in imaginal discs, analyzed by Optiprep density gradient centrifugation and WB. (F) Density of Hh lipid modification mutants (Hh^C85S^, Hh-N, Hh-N^C85S^), secreted into the hemolymph from the fat body (FB) with *lpp*-GAL4, analyzed by Optiprep density gradient centrifugation and WB.

Since both human and *Drosophila* tissue culture cells release Hh proteins in both lipoprotein-associated and non-associated forms, we wondered which forms might be found in vivo. Interestingly, we noted that Hh proteins are present in animal circulation; Shh in 100,000 *g* supernatants of human serum is present in density gradient fractions that contain LDL/HDL (Figure S3A). Likewise, *Drosophila* Hh is present in hemolymph, the insect body fluid (Figure 2A). The *Drosophila* hemolymph represents a convenient system to study in vivo Hh secretion, because the secreted form is readily accessible and produced in the presence of all potentially relevant extrinsic factors. Like Hh secretion in imaginal discs, Hh secretion into circulation requires Dispatched (Disp) (Figure 2B) [25]. However, imaginal discs are not a major source of hemolymph Hh; RNAi-mediated Hh knock-down in discs does not affect hemolymph Hh levels (Figure S3B). Neither is Hh produced in the larval fat body (Figure S3C), the major source of *Drosophila* lipoproteins [26]. The source and function of circulating Hh will be reported elsewhere (Rodenfels et al., manuscript in preparation). To ask whether hemolymph Hh circulated on lipoproteins, we fractionated third instar larval hemolymph on density gradients and probed for Hh and for apoLII, a scaffolding apolipoprotein of Lpp. All detectable Hh co-fractionates with apoLII, suggesting that hemolymph Hh is secreted on Lpp under normal circumstances (Figures 2C and S3D).

Our observation that a variety of mammalian cell types can secrete Shh on lipoproteins led us to wonder how general this capacity might be in *Drosophila* tissues. Since the hemolymph allows unambiguous detection of secreted Hh, we used the GAL4 system to induce ectopic expression of Hh in different tissues and examined the secretion form present in the hemolymph. While imaginal discs do not normally contribute to hemolymph Hh, driving Hh overexpression in discs raises the level of Lpp-associated Hh in circulation (Figure 2D,E). Thus, discs can secrete Lpp-associated Hh, consistent with previous results [12]. Furthermore, the fat body and midgut can secrete high levels of Lpp-associated Hh into the hemolymph in a Disp-dependent manner (Figure S4A–D). The vast majority of overexpressed wild-type Hh cofractionates with Lpp, even at the earliest time points after inducing expression (Figure S4E). Thus, a wide variety of cell types, including disc epithelial cells as well as unpolarized fat body cells, can release Hh in association with lipoproteins.

To ask which lipid modifications were required for association of *Drosophila* Hh with Lpp, we investigated Hh lipid modification mutants—Hh^C85S^, which lacks palmitate; Hh-N, which lacks sterol; and Hh-N^C85S^, which lacks both modifications. We ectopically expressed these different lipid mutant variants in the fat body, using the fat body-specific driver *lpp*-GAL4 (Figure S4F,G). Density gradients show that Hh^C85S^ is released entirely on Lpp (Figure 2F). Hh-N is released predominantly on Lpp, although small amounts are observed in high-density fractions. In contrast, Hh-N^C85S^ is present entirely in high-density fractions. This indicates that most Hh secreted by the fat body is both palmitoylated and sterol-modified and that either lipid modification suffices for Lpp association.

To ask whether Lpp was needed for secretion of wild-type Hh to circulation, we examined the effects of RNAi-mediated Lpp knock-down. Analysis of hemolymph in density gradients revealed that Hh is still secreted into the hemolymph when Lpp levels are strongly reduced. While some Hh co-fractionates with residual Lpp, most is present in dense fractions that do not contain apoLII, a scaffolding protein of Lpp (Figure 2C). Reduced sterol levels cannot account for the appearance of high-density Hh, because dietary sterol depletion does not produce this phenotype (Figure S3E,F). In addition to Lpp, *Drosophila* hemolymph contains two minor lipoproteins of higher density, Lipid Transfer Particle (LTP) and Crossveinless-d (Cv-d) [26]. However, most hemolymph Hh secreted in Lpp RNAi larvae fractionates at even higher density than LTP or Cv-d (Figure S3G). This high-density form of Hh therefore does not correspond to LTP or Cv-d-associated Hh; rather, it is a lipoprotein-independent form. Thus, *Drosophila* Hh can be secreted into the hemolymph as both Lpp-associated Hh and as a lipoprotein-independent form.

### Lipoprotein-Free Hh and Shh Are Secreted as Non-Sterol-Modified Dimers/Monomers

**Figure 3:**
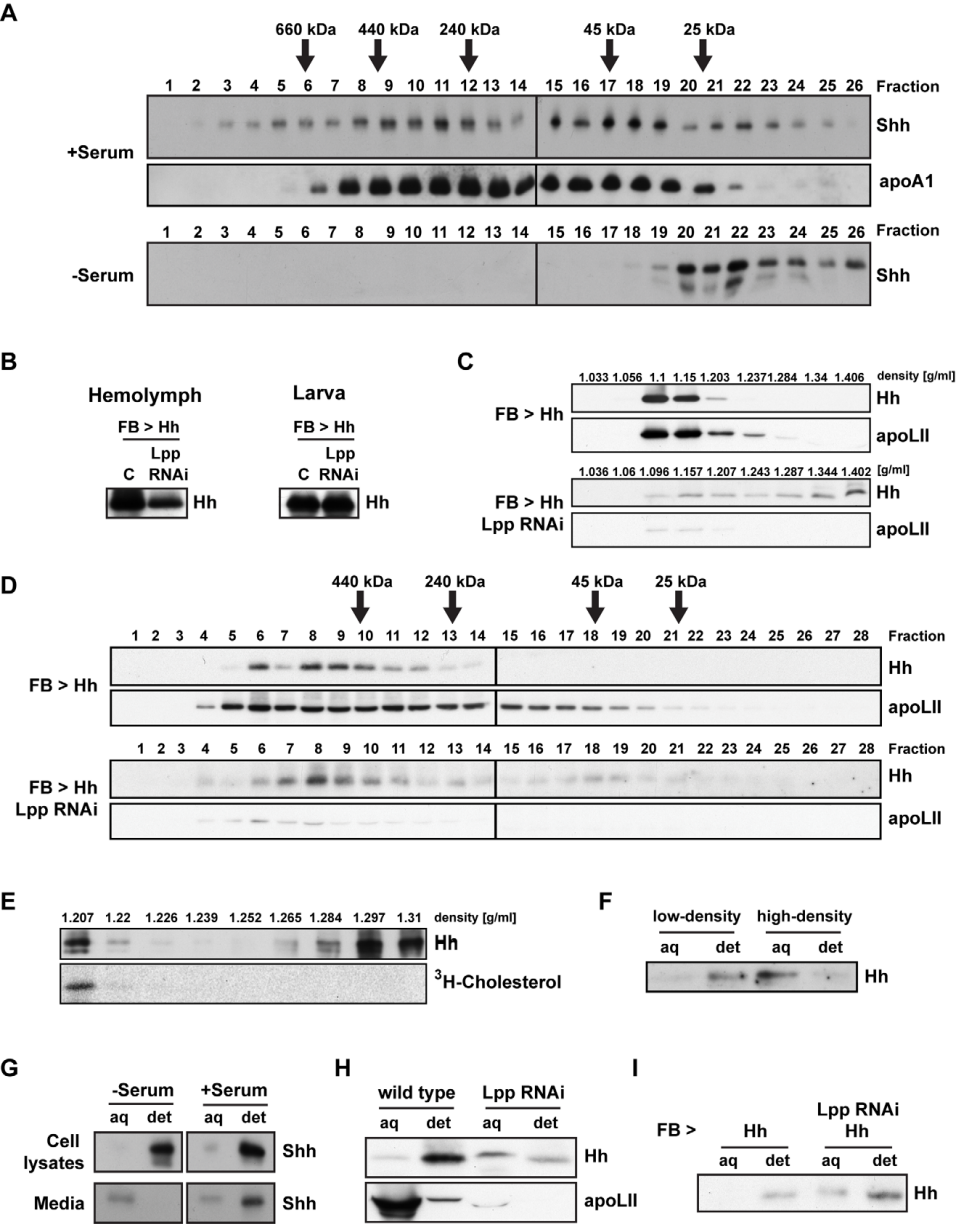
Molecular properties of lipoprotein-associated and lipoprotein-free Hh proteins. (A) Size of lipoprotein-associated and lipoprotein-free Shh secreted from HeLa cells grown in the presence or absence of FBS. Supernatants were fractionated by gel filtration chromatography on a Superdex 200 column, and fractions analyzed by WB. Molecular weight standards are indicated. Note that large mammalian lipoproteins run in the void volume of these columns. (B) Hemolymph Hh levels in *Drosophila* larvae ectopically expressing Hh in the fat body, with or without concomitant Lpp RNAi, analyzed by WB. (C) Density of hemolymph Hh produced in the fat body of control or Lpp RNAi larvae, analyzed by Optiprep density gradient centrifugation and WB. (D) Size of Lpp-associated and Lpp-free hemolymph Hh ectopically produced in the fat body of control or Lpp RNAi larvae, analyzed by gel filtration chromatography as described in (A). (E) Cholesterol modification status of low- and high-density forms of *Drosophila* Hh. Supernatant from Hh-producing S2 cells grown in the presence of FBS and ^3^H-cholesterol was fractionated by KBr density gradient centrifugation, and subsequently analyzed by both WB and in-gel fluorography. (F) Hydrophobicity of low- and high-density forms of Hh. Supernatants from Hh-producing S2 cells grown in the presence of serum were fractionated by KBr density gradient centrifugation; fractions 1 and 8 were analyzed by Triton X-114 phase separation and WB. (G–I) Hydrophobicity of Shh and Hh produced in the presence or absence of lipoproteins, assessed by Triton X-114 phase separation and WB. (G) Lysates and media from HeLa cells expressing Shh and grown in the presence or absence of FBS. (H) Hemolymph from control and Lpp RNAi larvae. (I) Hemolymph from control and Lpp RNAi larvae ectopically producing Hh in the fat body. Note that aqueous phase Hh/Shh has a lower electrophoretic mobility than detergent phase Hh/Shh.

We wondered how Hh and Shh were secreted independently of lipoproteins as high-density forms. Hh proteins have been proposed to assemble into high molecular weight multimers that interact via their lipid anchors [8]–[11]. Both Hh and Shh have also been observed in monomeric forms. We therefore used gel filtration chromatography to compare the sizes of different secreted forms of Hh and Shh. Shh secreted in the absence of lipoproteins elutes exclusively in low molecular weight fractions consistent with Shh monomers (Figure 3A). This suggests that the high-density form of Shh released by HeLa cells independently of lipoproteins is not multimeric; rather, it corresponds to free Shh. Shh from HeLa cells cultured in serum-containing medium is found both in fractions consistent with monomers and in high molecular weight fractions overlapping those containing the apolipoprotein apoA1 (Figure 3A). Thus, high molecular weight complexes may correspond entirely to lipoprotein-associated Shh.

To confirm that high-molecular weight pools correspond to the low-density form of Shh, we pooled different sizing column fractions and analyzed them by density gradient centrifugation. Indeed, Shh in size ranges above 240 kDa fractionates entirely at low density (Figure S5A). Conversely, Shh present in sizing column fractions consistent with monomers is found in high-density gradient fractions. Thus, HeLa cells produce at least two forms of Shh, a high molecular weight, lipoprotein-associated form and a monomeric form.

To characterize the size of the different secretion forms of *Drosophila* Hh in hemolymph, we generated them in larger amounts by overexpressing Hh in the fat body, alone or in combination with induction of Lpp RNAi (Figure 3B,C). Gel filtration chromatography revealed that in wild-type, Hh is present in high molecular weight complexes that are the same size as Lpp (Figure 3D). Examining these high molecular weight complexes in density gradients confirms that they correspond to low-density Hh (Figure S5B). Reducing Lpp levels causes the appearance of an additional pool of Hh that fractionates near the 45 kDa standard (Figures 3D and S5C). Thus, similar to HeLa cells, the *Drosophila* fat body can secrete Hh into the hemolymph as a high molecular weight Lpp-associated form, or as a low molecular weight complex (possibly dimers) independently of Lpp.

It seemed unlikely for the normally lipid-modified Hh proteins to be soluble as monomers or dimers. Therefore, we considered the possibility that this pool of Hh/Shh lacked lipid modification. To test this directly, we labeled Hh-producing S2 cells with ^3^H-cholesterol under conditions where they produced similar amounts of low-density and high-density forms. We subjected supernatants to density gradient centrifugation and analyzed each fraction both by Western blotting and in-gel fluorography. Western blotting detects the Hh N-terminal domain in both low- and high-density fractions, however only the low-density pool of Hh has incorporated radioactive cholesterol (Figure 3E). Thus, high-density Hh is not modified by sterol.

Sterol modification can also be assayed by partitioning into detergent and aqueous phases of Triton X-114 solutions [3],[4]. In this assay, sterol-modified Hh from low-density fractions of S2 cell supernatants partitions predominantly into the detergent phase (Figure 3F). In contrast, non-sterol-modified Hh in high-density fractions partitions almost exclusively into the aqueous phase. Triton X-114 phase partitioning of Hh/Shh variants genetically engineered to lack different lipid moieties shows that sterol is necessary and sufficient for partitioning into the detergent phase, regardless of the presence of palmitate (Figure S5D,E). We therefore used Triton X-114 partitioning to assess whether the high-density forms of Hh/Shh produced by other cells were sterol-modified. Shh secreted by HeLa cells in the absence of lipoproteins partitions exclusively into the aqueous phase, suggesting that it is not sterol-modified (Figure 3G). In contrast, Shh secreted in the presence of lipoproteins partitions mostly into the detergent phase, suggesting that lipoproteins allow the release of sterol-modified Shh. The minority of Shh that partitions into the aqueous phase also migrates slightly more slowly than detergent phase Shh, consistent with the reduced mobility of Hh proteins that lack sterol moieties [3],[4]. To confirm that non-sterol-modified Shh in these supernatants corresponds to the monomeric/high-density pool, we subjected pools of size fractionated Shh to Triton X-114 phase partitioning. Indeed, while the high molecular weight pool partitions into the detergent phase, the low molecular weight pool partitions into the aqueous phase (Figure S5A). Thus, while the lipoprotein-associated pool of Shh is sterol-modified, the monomeric pool likely is not. Taken together, these data suggest that HeLa cells release only monomeric, non-sterol-modified Hh in the absence of lipoproteins, but can also release sterol-modified Shh when lipoproteins are added.

To assess the sterol modification of Lpp-associated and high-density *Drosophila* Hh forms secreted into the hemolymph, we performed similar Triton X-114 phase partitioning experiments on wild-type and Lpp RNAi hemolymph. Lpp-associated Hh partitions to the detergent phase, indicating it is sterol-modified (Figure 3H,I). In contrast, Hh isolated from Lpp RNAi hemolymph, which comprises Lpp-associated and Lpp-free pools, is found in both detergent and aqueous phases. Furthermore, aqueous phase Hh migrates more slowly than detergent phase Hh, similar to non-lipid-modified Hh mutants (Figure S5F). Thus, Lpp-associated Hh present in the hemolymph is modified by sterol and Lpp-free Hh likely is not.

Taken together, these data suggest that lipoproteins promote release of sterol-modified Hh/Shh, whereas the Hh/Shh forms that are secreted independently of lipoproteins appear to be non-sterol-modified. The non-sterol-modified form is indistinguishable in electrophoretic mobility and hydrophobicity from Hh-N/Shh-N, which have been genetically engineered to lack sterol modification. This suggests that their C-terminal sequence is very close or identical to that of Hh-N/Shh-N; however, mass spectrometric analysis will be required to determine it precisely. We hereafter denote these endogenously produced Hh proteins that lack sterol Hh-N*/Shh-N*.

### *Drosophila* Imaginal Discs Release Both Lpp-Associated Hh and Hh-N*

**Figure 4:**
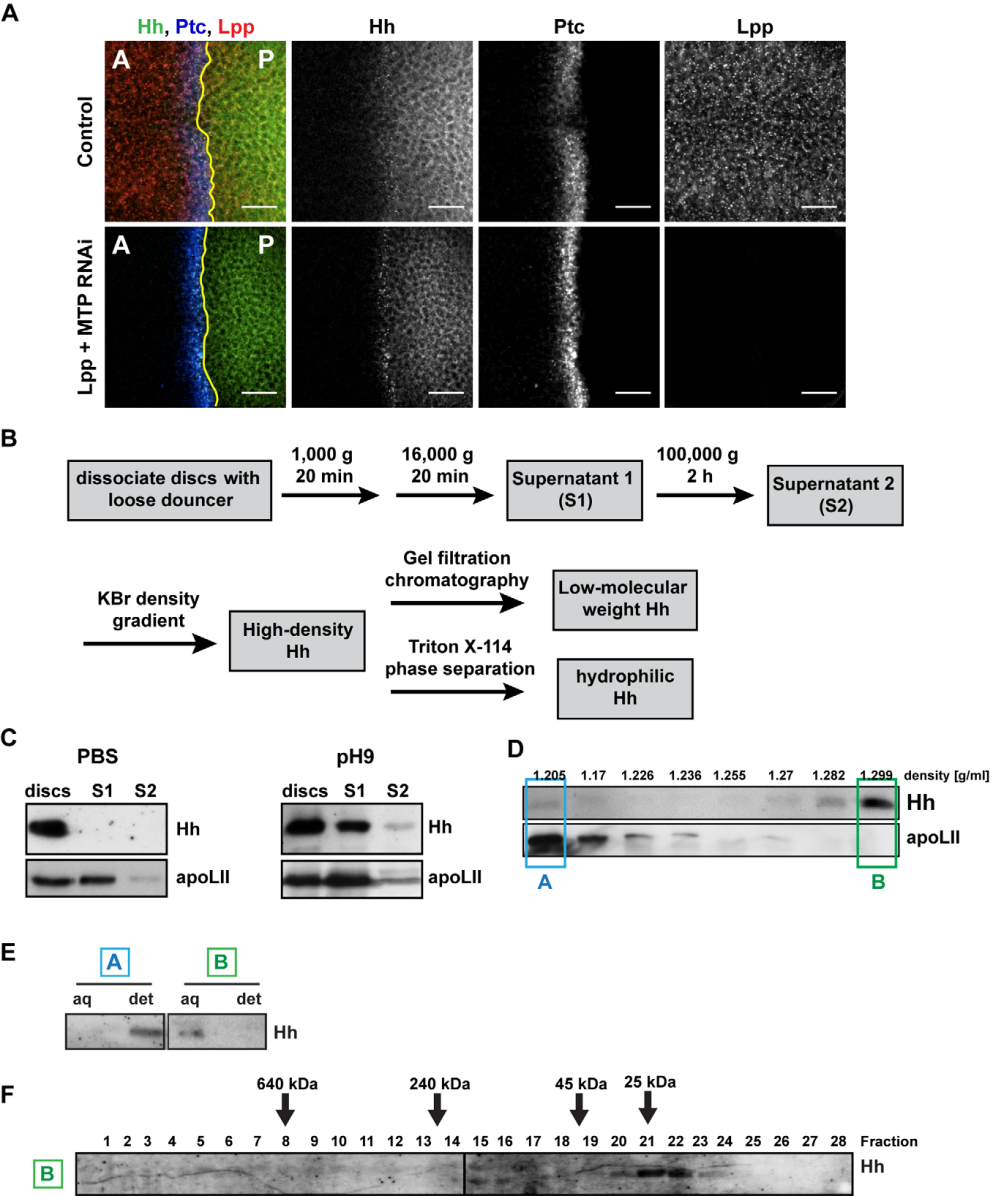
Imaginal discs produce Hh-N*. (A) Immunofluorescence (IF) of wing imaginal discs of MTP/Lpp co-RNAi larvae, stained for Hh, Lpp, and Ptc. A denotes the anterior compartment, P the posterior compartment; yellow lines indicate the compartment boundary. Scale bar = 20 µm. (B) Experimental scheme for the purification of Hh-N* from imaginal discs. Samples for experiments shown in (D–F) were prepared in 100 mM Na_2_CO_3_, pH 9. (C) WB showing the levels of Hh recovered from imaginal discs with PBS or 100 mM Na_2_CO_3_, pH 9 after 16,000 *g* (S1) and 100,000 *g* (S2) centrifugation steps. Sample amount in each lane corresponds approximately to the imaginal discs of two third instar larvae. (D) Density of Hh in 100,000 *g* supernatant of imaginal discs dissociated in pH 9 buffer, analyzed by KBr density gradient centrifugation and WB. Hh is present mainly in high-density fractions; in some experiments, minor amounts of Hh can also be detected in low-density fractions. (E) Hydrophobicity of low-density and high-density Hh recovered from imaginal discs, analyzed by Triton X-114 phase separation and WB. (F) Size of high-density Hh recovered from imaginal discs, analyzed by gel filtration chromatography and WB.

Although wing imaginal discs can release Hh on Lpp, our previous results suggested that they also must release Hh in a Lpp-independent form; Lpp RNAi does not prevent Hh secretion in wing discs, but reduces its signaling range [12]. Because Shh can be released on the different mammalian lipoprotein classes, we first considered the possibility that other *Drosophila* lipoproteins might mobilize Hh in wing discs. To more stringently remove lipoproteins, we performed simultaneous RNAi against both Lpp and Microsomal Triglyceride Transfer Protein (MTP). MTP is required for assembly of the two lipoproteins Lpp and LTP [26]. Blocking both Lpp and LTP secretion causes defects similar to Lpp RNAi alone; released Hh is detected over a shorter distance in the anterior compartment, and its signaling range is correspondingly reduced (Figure 4A). Furthermore, knock-down of either LTP or Cv-d alone does not affect Hh signaling in the wing disc (Figure S6A,B). This argues that Hh secreted by wing discs upon Lpp RNAi represents a lipoprotein-independent form. We therefore considered whether discs might normally release a non-sterol-modified form of Hh.

Although Hh overexpression in imaginal discs increases the amount of Lpp-associated Hh in the hemolymph, it does not result in detectable levels of high-density hemolymph Hh (Figure 2D). Therefore, if discs do secrete this form of Hh, it may be more tightly associated with cells or the extracellular matrix. We noted that the N-terminal signaling domains of *Drosophila* and mammalian Hh proteins have an unusually high pI (8.45–8.75) and thus should be positively charged at physiological pH. To disrupt possible ionic interactions between Hh proteins and imaginal disc tissue, we dissociated disc cells under either mildly alkaline (pH 9) or high salt (0.5 M NaCl) conditions. These conditions do not disrupt sterol linkage; even pH 10 is insufficient to cleave the ester bond between Hh and cholesterol (Figure S6C). To enrich for low molecular weight forms of Hh, we prepared 100,000 *g* supernatants; a large fraction of Lpp is pelleted at 100,000 *g*, along with small membrane fragments and exovesicles (Figure 4B,C) [27]. Dissociation of discs at pH 9 dramatically increased recovery of Hh in 100,000 *g* supernatants, compared to dissociation in PBS. Density gradient centrifugation of these supernatants shows that most Hh is of high density, consistent with depletion of membrane and Lpp-associated pools (Figure 4D). Similarly, high salt conditions allow the recovery of high-density Hh in 100,000 *g* supernatants (Figure S6D). Triton X-114 phase partitioning indicates that high-density Hh is not sterol-modified (Figure 4E). Smaller amounts of Hh are sometimes detectable in low-density fractions, which also contain residual Lpp; Triton X-114 phase separation confirms that this pool of Hh is sterol-modified. On sizing columns, high-density Hh from imaginal disc supernatants is found in fractions consistent with monomers (Figure 4F). Taken together, our data suggest that imaginal discs can release Hh in two forms: one is sterol-modified and Lpp-associated, the other is neither sterol-modified nor Lpp-associated. We obtained similar low- and high-density pools of Hh from *Drosophila* embryos dissociated at mildly basic pH or in high salt, suggesting that release of these two Hh forms is widespread (Figure S7). The non-sterol-modified form appears to be monomeric. Note that the buffer conditions we used do not disrupt lipoprotein particles, or the association of Hh with lipoproteins (unpublished data). However, we cannot rule out that these buffer conditions might disrupt complexes of Hh and other proteins, if these required ionic interactions for their integrity.

### *Drosophila* Lpp-Associated Hh Stabilizes Ci155 But Does Not Activate Target Gene Expression

**Figure 5:**
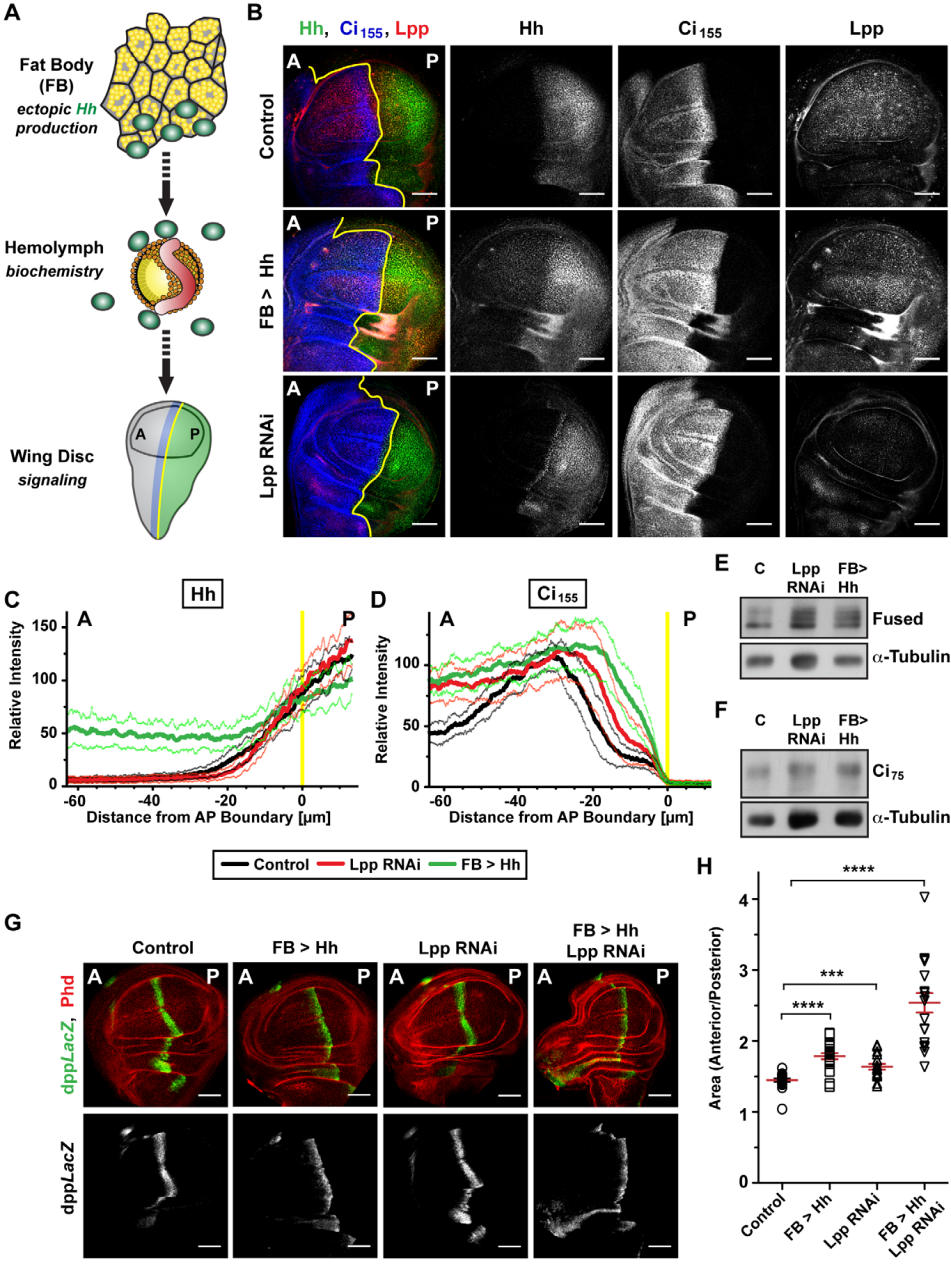
Signaling properties of Lpp-associated Hh and Hh-N*. (A) Cartoon depicting the fat body to wing disc signaling assay of secreted Hh. (B) IF of wing discs from larvae secreting Hh from the fat body, and Lpp RNAi larvae, stained for Hh, Ci_155_, and Lpp. Scale bar = 50 µm. In all wing discs, A denotes the anterior compartment, P the posterior compartment; yellow lines indicate the compartment boundary. Scale bar = 50 µm. (C and D) Quantification of (C) Hh and (D) Ci_155_ staining of wing discs shown in (B). Translucent lines indicate ±SD (*n* = 12). (E) Phosphorylation status of Fused in wing discs of larvae secreting Hh from the fat body and Lpp RNAi larvae, analyzed by WB. (F) Ci_75_ repressor levels in wing discs of larvae secreting Hh from the fat body, and Lpp RNAi larvae, analyzed by WB. (G) IF of wing discs from larvae secreting Hh or Hh-N* from the fat body, stained for *dpp*LacZ and, to mark cell boundaries, with phalloidin. Hh-N* was generated by expressing Hh in the fat body of Lpp RNAi animals. Scale bar = 50 µm. (H) Wing disc anterior to posterior compartment ratio of larvae secreting Hh or Hh-N* from the fat body and Lpp RNAi larvae. Error bars indicate ± SEM (*n* = 20). ***p<0.0005; ****p<0.00005.

We wondered whether the signaling activity of Lpp-associated Hh and Hh-N* might differ. To test this, we sought a method to expose wing discs specifically to each of these forms. To achieve this, we ectopically produced different forms of Hh in the fat body and assayed their activity in wing discs (Figure 5A). This method has the advantage that the form and amount of Hh to which the wing disc is exposed can be defined by analyzing the hemolymph. We monitored pathway activity by three different methods. We performed Western blotting against the Hh pathway components Ci and Fused. During pathway activation, Fused becomes multiply phosphorylated by different mechanisms; the extent of its phosphorylation correlates with pathway activity and can be monitored by electrophoretic mobility shifts [28]. We further used immunostaining to detect the distribution of full-length Ci_155_ and target gene expression (*en*, *engrailed*; *col*, *collier*; *dpp*, *decapentaplegic*) in the wing pouch. Hh endogenously produced in the posterior compartment of the wing disc signals to anterior cells close to the compartment boundary. To distinguish the effects of Hh provided exogenously through the hemolymph, we assayed signaling in anterior regions far from the AP boundary, which are normally not exposed to Hh. We also compared the relative sizes of the anterior and posterior compartments; overexpressing Hh in the wing disc causes anterior overgrowth, presumably because of increased production of Dpp [29].

To ask whether raising the amount of circulating Lpp-associated Hh could elevate Hh levels in wing discs, we used different GAL4 drivers to increase hemolymph Hh—*lpp*-GAL4, which increases circulating Hh levels dramatically, and *npc1b*-GAL4, which increases Hh levels more moderately (Figure S4A). The moderate amounts of hemolymph Hh produced by *npc1b*-GAL4 do not increase Hh staining or affect Hh signaling in the wing disc (Figure S8A–C). Thus, the even lower amount of Hh normally present in circulation is unlikely to influence the shape of the wing disc Hh gradient. In contrast, the much higher circulating Lpp-associated Hh levels induced by *lpp*-GAL4 raise Hh staining throughout the anterior compartment of the wing disc to levels normally observed only near the AP boundary (Figure 5B,C). Thus, this condition provides Lpp-associated Hh to the whole wing disc in relevant amounts, allowing us to specifically assess its signaling activity.

**Figure 6:**
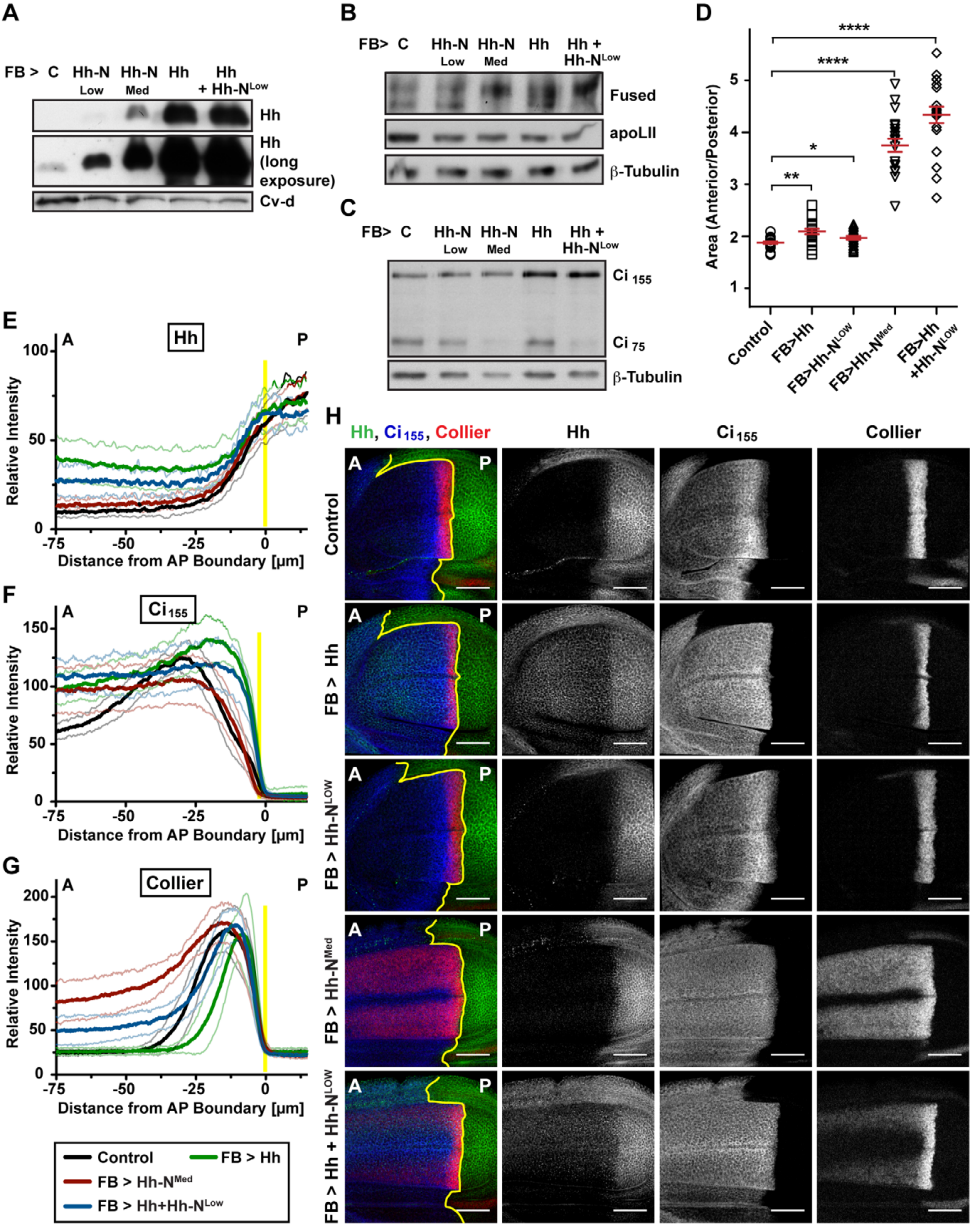
Signaling properties of Hh-N. (A) Hemolymph Hh levels of larvae secreting Hh, Hh-N^Med^, Hh-N^Low^, or Hh+Hh-N^Low^ from the fat body, analyzed by WB. (B) Phosphorylation status of Fused in wing discs from larvae secreting different combinations of Hh and Hh-N from the fat body, analyzed by WB. (C) Levels of Ci repressor (Ci_75_) and Ci_155_ (full-length) in wing discs of larvae secreting different combinations of Hh and Hh-N from the fat body, analyzed by WB. (D) Wing disc anterior to posterior compartment ratio of larvae secreting different combinations of Hh and Hh-N from the fat body. Error bars indicate ± SEM (*n* = 20). *p<0.05; **p<0.005; ***p<0.0005; ****p<0.00005. (E–G) Quantification of (E) Hh, (F) Ci_155_, and (G) Collier staining of wing discs shown in (H). Translucent lines indicate ± SD (*n* = 12). (H) IF of wing discs from larvae secreting different combinations of Hh and Hh-N from the fat body, stained for Hh, Ci_155_, and Collier. A denotes the anterior compartment, P the posterior compartment; yellow lines indicate the compartment boundary. Scale bar = 50 µm.

Lpp-associated Hh stabilizes Smo (Figure S8D) and Ci_155_ (Figure 5B,D) throughout the wing pouch and increases phosphorylation of Fused (Figure 5E). However, Lpp-associated Hh does not increase transcription of *dpp* (Figure 5G), *en* (Figure S8F,G), or *col* (Figure 6G,H), and causes barely detectable anterior overgrowth (Figure 5H). This is surprising, because Hh present at similar levels in the normal wing disc gradient activates these target genes. Western blotting reveals that Lpp-associated Hh, although it increases the amount of full-length Ci_155_, does not reduce levels of the repressor Ci_75_ (Figures 5F and S8D). Thus, Lpp-associated Hh has only a subset of the activities of Hh normally released by wing disc cells. The effects of Lpp-associated Hh are remarkably similar to those of Lpp RNAi—increased phosphorylation of Fused and stabilization of Ci_155_ without reduction of Ci_75_ or activation of target genes (Figure 5) [13]. This suggests that Lpp-associated Hh does not promote Ci_155_ activation or block its processing to the repressor form; rather, it specifically stabilizes inactive full-length Ci_155_. We note that these high levels of Lpp-associated Hh actually repress *en* and *col* expression in the anterior compartment (Figures 6G,H and S8F,G), suggesting that they compete with Hh produced locally by posterior compartment cells. This is consistent with the idea that wing discs normally make not only Lpp-associated Hh but also a Lpp-independent form.

### *Drosophila* Hh-N* and Hh-N Can Activate Target Gene Expression

As the effect of Lpp-associated Hh on the Hh pathway is confined to Ci_155_ stabilization, we wondered whether Hh-N*, which is endogenously produced by imaginal discs, might exert additional effects leading to target gene transcription. To address this, we reduced Lpp-mediated Ci_155_ degradation and simultaneously exposed wing discs to additional Hh-N* provided by the hemolymph. We accomplished this by concomitant expression of Lpp RNAi and Hh in the fat body. Under these conditions, the hemolymph contains moderate levels of Hh-N*; some Hh is also detected on residual Lpp (Figure 3C). The total amount of Hh present in the hemolymph is less than when Hh is ectopically produced by otherwise normal fat bodies (Figure 3B), and it accumulates at lower levels in the wing disc (Figure S9A,B). However, Hh produced by the fat body in Lpp RNAi animals induces ectopic expression of *dpp* in some regions of the wing disc (Figure 5G) and causes strong anterior overgrowth (Figure 5H). This suggests that Hh-N* can induce transcription of target genes, at least when Ci_155_ is stabilized by Lpp RNAi. The fact that even larger amounts of Lpp-associated Hh do not produce these effects suggests that Hh-N* is either more potent or that its signaling activity is different.

Since our biochemical data indicated that Hh-N* lacked sterol modification, we wondered whether its activity might be mimicked by Hh-N, which is genetically engineered to lack sterol modification. The signaling activity of Hh-N/Shh-N has been investigated in different systems including wing discs. Their activity compared to wild-type proteins has been controversial—they have been proposed to signal over both longer and shorter ranges than the wild-type form [10],[30],[31]. However, it has been difficult to determine the concentration of secreted Hh-N or sterol-modified Hh to which tissue is exposed in vivo. To compare the signaling activity of Hh-N to that of wild-type Hh at definable concentrations, we expressed each protein in the fat body and first compared their levels in the hemolymph. We used two different Hh-N expression constructs (Hh-N^Med^ and Hh-N^Low^) to drive Hh-N expression. Both produce much lower concentrations of circulating Hh than the wild-type Hh expression construct (Figure 6A). The weak Hh-N^Low^ does not increase wing disc Hh staining detectably when expressed in the fat body. Driving Hh-N^Med^ in the fat body does increase Hh levels in the wing disc, but to a much lesser extent than expression of wild-type Hh (Figures 6E and S10A,B).

We next compared how these defined amounts of Hh-N and wild-type Hh derived from the fat body influenced signaling in the wing disc. In striking contrast to wild-type Lpp-associated Hh, which only causes Ci_155_ stabilization and a moderate increase in Fused phosphorylation, these moderate levels of Hh-N produced from Hh-N^Med^ elicit the full spectrum of Hh responses; Fused is extensively phosphorylated (Figure 6B), Ci_75_ levels are reduced (Figures 6C and S10E), and Ci_155_ accumulates (Figure 6F,H). Furthermore, target gene expression is activated throughout the wing pouch (Figures 6G,H and S10A–C), and wing discs strongly overgrow anteriorly (Figure 6D). The minor amounts of Hh-N produced by Hh-N^Low^ do not affect target gene activation. However, when combined with wild-type Hh, they suffice to extensively phosphorylate Fused (Figure 6B), deplete Ci_75_ (Figures 6C and S10F), activate target gene expression (Figures 6G,H and S10G), and cause anterior overgrowth (Figure 6D). Thus, Hh-N has a qualitatively different signaling activity from wild-type Hh and can activate transcription of target genes. A possible explanation for this is that Hh-N has features of both wild-type Lpp-associated Hh and Hh-N*. It can associate with Lpp, but lacks sterol modification and is partially released as a high-density form. This raises the possibility that the combination of these Hh forms is more powerful that either one alone. Consistently, even otherwise inactive amounts of Hh-N suffice to induce target gene expression if Ci_155_ is stabilized by wild-type Lpp-associated Hh.

### Lipoprotein-Associated Shh Reverses Pathway Inhibition by Mammalian Lipoproteins

**Figure 7:**
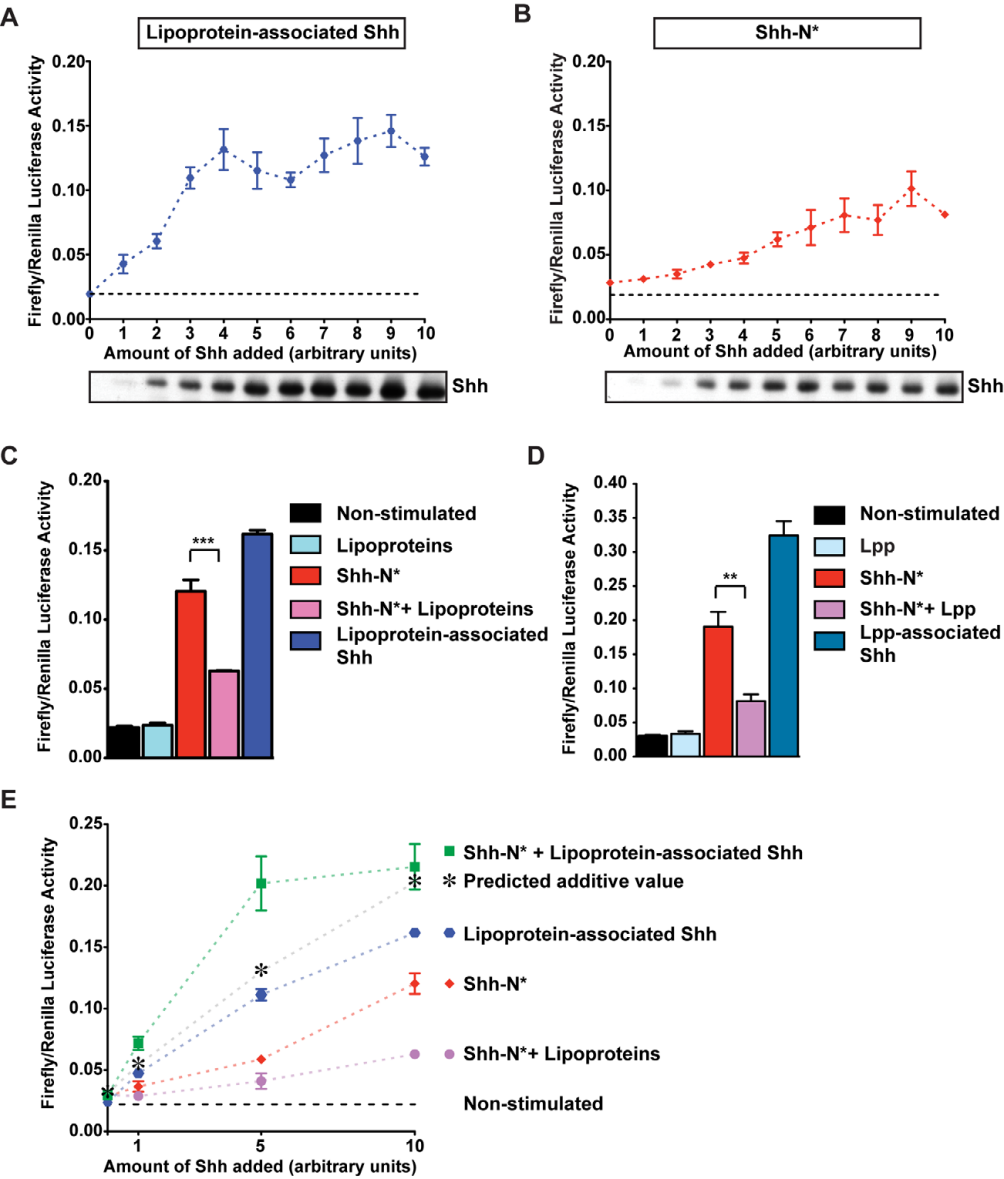
Signaling properties of lipoprotein-associated Shh and Shh-N* in Shh-LIGHT2 cells. (A, B) Concentration-dependent signaling activity of (A) lipoprotein-associated Shh and (B) Shh-N*. Lipoprotein concentration in (A) was kept constant, and only the fraction carrying Shh increased. Shh and Shh-N* levels used for signaling assays were assessed by WB. (C,D) Shh pathway activity in cells stimulated by Shh-N* in the absence or presence of lipoproteins, or cells stimulated with lipoprotein-associated Shh. Lipoproteins, where added, were kept at a constant level. (C) Mammalian lipoproteins, (D) *Drosophila* Lpp. (E) Synergistic signaling activity of Shh-N* and lipoprotein-associated Shh. Shh-N* and lipoprotein-associated Shh were applied to cells alone or in combination. Predicted additive values represent the combined activity of lipoprotein-associated Shh and Shh-N* in the presence of lipoproteins, minus the basal assay activity measured in unstimulated cells. Note that the same batch of samples was used for assays shown in (A) and (B). For (A–E), error bars indicate ± SD (*n* = 3; **p<0.005; ***p<0.0005) of one representative experiment. Experiments were repeated at least twice.

To investigate the signaling abilities of lipoprotein-associated Shh and Shh-N*, we assayed their activities in Shh-LIGHT2 cells. These cells harbor a luciferase reporter sensitive to Gli repressors and activators [32],[33]. We isolated lipoprotein-associated Shh from supernatants of cells grown with serum by density centrifugation. We prepared Shh-N* from supernatants of cells grown in serum-free medium. We added increasing amounts of either lipoprotein-associated Shh (Figure 7A) or Shh-N* (Figure 7B) to Shh-LIGHT2 cells in serum-free conditions. For lipoprotein-associated Shh, lipoprotein concentrations were kept constant; only the fraction of lipoproteins carrying Shh was varied. Both forms of Shh increase reporter activity in a concentration-dependent fashion (Figure 7A,B). To ask whether lipoproteins repress signaling by Shh-N*, we added both Shh-N* and purified lipoproteins to Shh-LIGHT2 cells. Under these conditions, the vast majority of Shh-N* remains unassociated with lipoproteins (Figure S11A). Reporter activation by Shh-N* is strongly reduced by lipoproteins in a dose-dependent manner (Figure S11B). In contrast, even much higher amounts of lipoproteins do not repress signaling by lipoprotein-associated Shh (Figures 7C and S11B). Thus, mammalian lipoproteins repress signaling by lipoprotein-free Shh-N*. However, binding of Shh to lipoproteins blocks their inhibitory action—similar to the situation in *Drosophila*.

This similarity led us to wonder whether the inhibitory molecules in lipoproteins were conserved. To test this, we asked whether *Drosophila* Lpp could inhibit signaling in Shh-LIGHT2 cells. Indeed, Lpp strongly represses signaling by Shh-N*. In contrast, Shh associated with Lpp signals efficiently (Figure 7D). Thus, the inhibitory activity of lipoproteins on the Hh pathway is conserved between *Drosophila* and mammals. Lipoprotein-associated Shh can overcome this inhibition, but Shh-N* cannot.

We wondered whether Shh-N* might signal more efficiently if lipoprotein-mediated inhibition was blocked by lipoprotein-associated Shh. We therefore assayed their activity in Shh-LIGHT2 cells alone and in combination. We chose concentrations of Shh either in the linear response range or at saturation concentrations (see Figure 7A,B). While Shh-N* signals only weakly in the presence of lipoproteins, adding Shh-N* together with lipoprotein-associated Shh causes a more than additive increase in reporter activity at nonsaturating concentrations of Shh (Figure 7E). This suggests that lipoprotein-associated Shh and Shh-N* may increase pathway activity synergistically.

## Discussion

A variety of different mechanisms have been proposed to account for the release and spread of dually lipid-modified Hh proteins [6],[34]. In mammalian systems, secreted Hh proteins have been observed in high-molecular weight complexes and on exovesicles [7]–[11]. Monomeric forms have also been detected, but how a doubly lipid-modified protein could remain soluble as a monomer has never been explained. In *Drosophila*, association with the insect lipoprotein Lpp promotes long-range Hh signaling, but Lpp knock-down experiments suggested that at least one shorter range form must also exist [12]. This apparent diversity in release mechanisms raises the question of how different forms of Hh act on cells. In this work, we establish that mammalian Shh can be released on lipoproteins, like its *Drosophila* counterpart, and we identify and biochemically characterize a second conserved release form that is monomeric/dimeric and non-sterol-modified (Hh-N*/Shh-N*). We use both mammalian signaling assays and in vivo experiments in *Drosophila* to distinguish their effects on the Hh pathway and show that they signal synergistically.

Our studies of lipoprotein-mediated release of Shh were facilitated by the use of cell types (HeLa and Mia-PaCa2) that rely on extrinsic factors (in this case lipoproteins) to release sterol-modified Shh. Interestingly, it was recently shown that extrinsic addition of Scube to tissue culture cells also promotes release of lipid-modified Shh [35],[36]. Together, these studies highlight the emerging importance of non-autonomously acting factors in the release of sterol-modified Hh proteins. HEK293 and Bosc23 cells, which have often been used to study Shh release mechanisms, are able to release some sterol-modified Shh even in defined serum-free media [11]. It would be interesting to ask whether these differences might be accounted for by endogenous production of factors like Scube or apolipoproteins.

### Conserved Functions for Lipoproteins in the Hh Pathway

Our work in tissue culture shows that an evolutionarily conserved feature of lipoproteins is their ability not only to mobilize Hh proteins, but also to repress pathway activity. A unique function of lipoprotein-associated forms of Hh is to reverse this pathway inhibition. To what extent are lipoproteins important for the in vivo function of mammalian Hh proteins? Genetic studies have not highlighted obvious links between mammalian lipoprotein metabolism and the Hh pathway. However, it has been noted that loss of apoB-containing lipoproteins does produce a subset of phenotypes similar to those of Hh loss of function [37],[38]. Knockouts of individual members of the large family of apoA-like apolipoproteins are viable [39],[40]. However, since we have shown that Shh can be released on all human lipoprotein classes, redundancy might make a requirement for lipoproteins hard to identify genetically. Furthermore, phenotypic analysis may be confounded by the fact that lipoproteins exert both positive and negative effects on the Hh pathway.

Many different tissues produce lipoproteins and they are abundant in circulation and interstitial fluids [41]–[43]. While access to large lipoproteins such as VLDL may be limited by the permeability of the vasculature in some tissues, HDL is abundant in interstitial fluid. Even before development of the embryonic vasculature is complete, apoA1 can be readily detected in mouse limb buds (M. Swierczynska and S. Eaton, unpublished observation). Thus, it will be interesting to assess the relative contributions of different lipoproteins to Shh release and signaling in vivo during development and adult tissue homeostasis. Many tumors secrete Hh proteins, which they require for growth [23],[44]. Interestingly, lipoprotein levels are unbalanced in metabolic syndrome, which is associated with an increased risk of cancer development. Tumors promote both angiogenesis and vascular permeability and should have access to a broad range of lipoproteins [45]. Thus, our observation that lipoproteins enhance Shh release by the pancreatic cancer cell line Mia-PaCa2 [24] may be relevant to a broad variety of Shh-secreting tumors in vivo.

In addition to mobilizing Hh proteins locally, our work suggests that lipoproteins may also function to carry them into systemic circulation. While we do not address the physiological role of circulating Hh here, we demonstrate that *Drosophila* Hh produced in one organ can signal to another in an endocrine manner. Hh signaling is known to influence lipid metabolism in both mammals and flies [46]–[48], and Hh associated with circulating lipoproteins may be important in this regard. Perturbed lipoprotein metabolism is a widespread phenomenon in modern society and is associated with a wide variety of different tissue pathologies [49]. Our results raise the possibility that lipoprotein dysfunction might cause disease in part by altering Hh signaling.

### Release of Hh Proteins as Non-Sterol-Modified Forms

Our work also demonstrates that Hh proteins can be released independently of lipoproteins in a non-sterol-modified form (Hh-N*/Shh-N*). Autocatalytic processing of the full-length Hh protein to the active N-terminal domain involves covalent linkage to sterol [3],[4]. How could cells generate non-sterol-modified Hh-N*/Shh-N*? Hh-N*/Shh-N* might be generated enzymatically by unknown sterol esterases or proteases, or cleavage of the thioester bond by an alternative nucleophile. These proteins cannot lack significant portions of the N-terminal signaling domain; Hh-N*/Shh-N* are of slightly slower electrophoretic mobility than their sterol-modified counterparts, migrating similarly to Hh proteins from which cholesterol has been removed either chemically or genetically [3]. This distinguishes Shh-N* from Shh released by metalloprotease-mediated shedding, which is a truncated protein with faster electrophoretic mobility than lipid modified Shh [50]. Many studies have observed monomeric/dimeric forms of secreted Hh proteins [8],[11],[51], although whether these were sterol-modified was never established. This raises the possibility that the autonomous ability of cells to release non-sterol-modified forms is widespread.

### Complementary Functions of Lipoprotein-Associated and Non-Sterol-Modified Forms of Hh

The diversity of different forms of secreted Hh raises the interesting issue of whether they might affect signaling in different ways. For the first time, to our knowledge, we show here that at least two of these different forms (lipoprotein-associated Hh and Hh-N*) have qualitatively different effects on the Hh pathway. We show that the lipoprotein-associated forms of both *Drosophila* Hh and mammalian Shh can block the repressive effects of lipoproteins on Hh signaling. In vivo experiments in *Drosophila* reveal that lipoproteins and Lpp-associated Hh regulate a specific subset of signaling events. Lpp-associated Hh can increase Smo levels, partially phosphorylate Fused, and increase Ci_155_ levels. However, Lpp-associated Hh cannot reduce levels of the Ci_75_ repressor form or activate expression of target genes. Non-sterol-modified Hh-N* has a complementary function; extremely low levels of Hh-N that do not affect signaling at all by themselves combine with Lpp-associated Hh to complete Fused phosphorylation, reduce the levels of Ci_75_ repressor, and activate target gene expression. Signaling experiments in Shh-LIGHT2 cells show that lipoprotein-associated Shh can induce reporter transcription in this assay; interestingly, it can also synergize with Shh-N* to further increase pathway activity. Our findings in *Drosophila* suggest it will be interesting to assay for different effects of each Shh form on ciliary Smo trafficking and processing of Gli2 and Gli3.

In general, it will be important to understand how these forms act differently at the cellular level. We showed previously that Ptc regulates Lpp trafficking and proposed that Ptc represses Smo, in part by mobilizing an inhibitory lipid from Lpp particles [13]. Lipoprotein association may facilitate the access of Hh/Shh to this compartment, allowing it to efficiently block mobilization of lipoprotein lipids by Ptc. In contrast, non-lipoprotein-associated Shh/Hh, although they likely bind to Ptc, may interact with different co-receptors (e.g. Ihog and Cdo-family proteins, or LRP2) with distinct signaling functions 52,53.

Much Hh research has focused on the importance of its graded spatial and temporal distribution in patterning gene expression [54],[55]. Our data raise the possibility that tissue-patterning information might be contained, not only in the absolute amount of Hh but in the relative proportions of different Hh forms. These forms may spread through tissue differently, form differently shaped gradients, and interact with different receptor complexes. Thus, the Hh activity gradient may reflect the superimposition of gradients created by distinct Hh forms. In general, regulating the secretion form of Hh might be one way of generating a diversity of Hh responses in different tissues.

## Materials and Methods

### Mammalian Expression Plasmids

cDNA encoding human Shh in pCMV-XL5 vector was purchased from OriGene (SC300021). Shh^C24S^, Shh-N, and Shh-N^C24S^ were generated by PCR.

### Fly Stocks

The following stocks were used: *lpp*-GAL4 [56], *en105*-GAL4 [57], *hh*-GAL4, *tub*-GAL4, *myoIA*-GAL4 [58], *npc1b*-GAL4 [59], *hh^ts2^*, UAS-Hh [29], UAS-Hh-N^Low^, UAS-Hh^C85S^, UAS-Hh-N^C85S^ [60], *dispS037707* [25], wild type Oregon R, *tub*-GAL80TS, *dpp*LacZ, *hs-flp* (Bloomington), UAS-Dicer2, Cv-d RNAi (VDRC), Lpp RNAi [12], LTP RNAi, and MTP RNAi [26].

UAS-Hh-N^Med^ was generated by phiC31-mediated integration of UAS-Hh-N into the VK33 *attP* landing site [61].

### Antibodies

Antibodies used: Shh, Hh [57], apoLII, apoLI, Lpp [12],[57], Collier [62], Cv-d, apoLTPII [26], Ci 2A1 [63], Ptc, Smo, En, Fused (DSHB), apoA1, apoB (Calbiochem), apoA1 (Abcam), α-tubulin (Sigma), β-tubulin (Invitrogen), and β-Galactosidase (Promega).

### Human Lipoprotein Isolation

VLDL, LDL, and HDL were isolated from human serum (Sigma) essentially according to [64]. Lipoproteins were applied to HeLa cells at concentrations found in human serum.

### Mammalian Cell Culture and Transfection

HeLa cells were grown in DMEM with 10% fetal bovine serum (FBS, GIBCO), 50 U/ml penicillin, and 50 µg/ml streptomycin (GIBCO).

HeLa cells were transfected with plasmids encoding Shh variants using polyethylenimine (Polysciences) in OptiMem (Invitrogen), then switched to experimental media 4 h after posttransfection, and cultured for 48 h prior to sample collection.

To prepare Shh-N*, Shh-transfected HeLa cells were cultured in serum-free media (DMEM+1% Insulin-Transferrin-Selenium mixture (ITS-X, GIBCO)). Conditioned media were centrifuged at 1,000 *g*, 20 min, then concentrated using Amicon Ultra 10K (Millipore) for density gradient analysis and signaling assays. Controls were identically treated media from nontransfected cells. Note that centrifugation at higher speeds begins to pellet large apoB-containing lipoproteins (Figure S12A).

To analyze association of Shh with lipoproteins, transfected HeLa cells were cultured in DMEM supplemented with either 10% FBS, human lipoproteins (VLDL, LDL, or HDL in final concentrations similar to those found in human serum), or *Drosophila* larval hemolymph. Conditioned media were centrifuged at 1,000 *g*, 20 min. Prior to density centrifugation, media were concentrated using Amicon Ultra 10K. For signaling assays, lipoprotein-associated Shh was isolated by density centrifugation [65] and concentrated using Amicon Ultra 10K. Control lipoproteins were prepared from identically treated media from nontransfected HeLa.

MIA PaCa-2 cells (ATCCs) were grown in DMEM with 10% FBS, 2.5% Horse Serum (Sigma), 50 U/ml penicillin, and 50 µg/ml streptomycin. When confluent, cells were switched to DMEM supplemented with 1% ITS and either VLDL, LDL, or HDL (in final concentrations similar to those found in human serum) and cultured for 48 h prior to sample collection. Conditioned media were centrifuged at 1,000 *g*, 20 min, concentrated using Amicon Ultra 10K, and analyzed by density gradient ultracentrifugation.

Cell lysates for Western blotting (WB) were prepared with CelLytic M (Sigma) according to manufacturer instructions.

### Expression of Fly Transgenes and RNAi

Hh variants were produced in the fat body using the UAS-GAL4 system and the *lpp*-GAL4 driver. Lpp RNAi was induced as described [12], using *lpp*-GAL4. Experiments were performed 4 d after RNAi induction. Crosses were kept at 25°C. Because continuous expression of Hh-N^Med^ in the fat body causes larval lethality, we temporally controlled expression of Hh variants in experiments involving this construct using *lpp*-GAL4, *tub*-GAL80^TS^ at 29°C.

### Hemolymph Isolation

Hemolymph was prepared as described [26]. Briefly, hemolymph was collected from punctured third instar larvae in PBS, centrifuged for 30 min at 1,000 *g*, then for 30 min at 16,000 *g*. Note that higher centrifugation speeds pellet a large fraction of hemolymph proteins (see Figure S12B). Isolation of Lpp on preparative scales was performed as described [12].

### Size and Density Fractionation of Hh Proteins

Isopycnic density centrifugation in Optiprep gradients (Fresenius Kabi) and KBr gradients was performed as described [12],[57].

Proteins were separated according to size by gel filtration chromatography on a Superdex 200 PC 3.2/30 column (GE Healthcare). Running buffer: PBS, 0.001% NP-40. Fraction volume: 50 µl. Size standards: Thyroglobulin (660 kDa), Ferritin (450 kDa), Catalase (240 kDa), Ovalbumin (45 kDa), and Chymotrypsin (25 kDa).

Triton X-114 phase separation was performed essentially according to [66]. Note that in these experiments, scaffolding apolipoproteins partition into the aqueous phase, whereas lipoprotein-associated, sterol-modified Hh proteins partition into the detergent phase (see Figures 3H and S12C). This indicates that the 1% Triton X-114 solutions used in these experiments suffice to disassemble lipoprotein particles or extract lipid-modified proteins from them.

Samples were precipitated with methanol/chloroform, separated on 15% polyacrylamide gels, and analyzed by WB.

### Co-Immunoprecipitation

HeLa cells were transfected with either Shh or Shh-N^C24S^. At 4 h posttransfection, media were changed to DMEM+1% ITS supplemented with either VLDL, LDL, or HDL (in final concentrations similar to those found in human serum). Two days later, media were collected and cleared by centrifugation at 1,000 *g* for 20 min. Supernatants were diluted with Tris-HCL, pH 7.4, to a final concentration of 50 mM Tris. Samples were incubated overnight with Protein-G agarose (Roche) and antibodies targeting apoB (Calbiochem) or apoA1 (Abcam). Beads were washed several times with 50 mM Tris, pH 7.4+0.15 M NaCl, and precipitated proteins subsequently analyzed by WB.

### Radioactive Labeling

Labeling of *Drosophila* Hh with ^3^H-cholesterol was performed essentially according to [4]. S2 cells stably expressing Hh [67] were labeled for 24 h with 150 µCi [1,2,6,7-^3^H(N)]-cholesterol (American Radiolabeled Chemicals) in Schneider's medium (GIBCO) supplemented with 1% ITS and 1% FCS (Sigma). Supernatants were centrifuged for 30 min at 1,000 *g*, then for 30 min at 16,000 *g*, and subsequently fractionated by KBr density gradient centrifugation. Precipitated proteins of the different fractions were analyzed both by WB and in-gel fluorography.

### Purification of Hh-N* from *Drosophila* Imaginal Discs and Embryos

Everted heads of wandering third instar larvae, from which all organs except imaginal discs were removed, were used as a relatively pure source of imaginal discs. Embryos were collected for 12 h and subsequently dechorionated. Tissues were broken by a loose douncer using buffer conditions as indicated for the different experiments (alkaline: 100 mM Na_2_CO_3_, pH 9; high salt: 10 mM Tris, pH 8+0.5 M NaCl). Note that the pH of the alkaline buffer is too low to induce cleavage of the ester bond between sterol and Hh (Figure S6C). Cells and large membrane fragments were pelleted by centrifugation for 20 min at 1,000 *g*, and subsequently for 20 min at 16,000 *g*. Small vesicles, exosomes, and a large fraction of lipoproteins were pelleted by centrifugation for 2 h at 100,000 *g*. Resulting supernatants were fractionated by KBr density gradient centrifugation. Indicated gradient fractions were subsequently fractionated by gel filtration chromatography on a Superdex 200 10/300 GL column (GE Healthcare). Running buffer: PBS, 0.001% NP-40. Indicated fractions from density gradients and sizing columns were further analyzed by Triton X-114 phase separation. For experimental overview of Hh-N* purification, see also Figures 4B and S7A.

### Shh Activity Assay

Shh-LIGHT2 cells [33] were maintained in DMEM+10% FBS, 150 µg/ml zeocin (Invitrogen), and 400 µg/ml G418 (Invitrogen). 24 h prior to assay, cells were plated at 10^5^/well in 96-well plates. Cells were then switched to DMEM with 1% ITS-X and supplemented with different combinations of Shh-N*, Lipoprotein-associated Shh, Lpp-associated Shh, bovine, or *Drosophila* lipoproteins. Luciferase activity was assayed in cell lysates after 24 h, as instructed by the manufacturer (Dual Glo Luciferase Assay, Promega).

### Tissue Staining

Wing disc staining was performed as described [12].

## Supporting Information

Figure S1 Shh is secreted in lipoprotein-associated and lipoprotein-free forms. (A) Shh levels in cell lysates from HeLa cells transfected with human Shh, grown in serum-free medium or in the presence of 10% FBS, analyzed by Western blotting (WB). (B) Density of different Shh lipid modification mutants. Supernatants from HeLa cells transfected with Shh, Shh^C24S^, Shh-N, or Shh-N^C24S^ and grown in the presence of FBS were analyzed by Optiprep density gradient centrifugation and WB. Note that it is difficult to determine whether palmitate might suffice for lipoprotein association, since mammalian tissue culture cells overexpressing Shh do not palmitoylate it efficiently [5],[69]. (C) WB of Shh secretion time course. HeLa cells were transfected with Shh and grown in serum-free medium. 24 h after transfection, medium was replaced by fresh medium supplemented with 10% FBS; supernatants were collected after the indicated periods of time, and equal volumes analyzed by Optiprep density gradient centrifugation. Colors indicate fractions corresponding to bovine Very Low-, Low-, and High-Density Lipoproteins (VLDL, LDL, and HDL) [68]. (D) Immunoprecipitation of different human lipoprotein classes from HeLa cell supernatants. ApoB was detected by Coomassie staining of gels; apoA1 was detected by WB. The same samples were analyzed for co-immunoprecipitated Shh in Figure 1E.
(TIF)

Figure S2 Hh proteins can associate with different human and *Drosophila* lipoproteins. (A) Shh levels in supernatants and cell lysates derived from MIA PaCa-2, grown in serum-free medium with or without addition of different human lipoproteins. Equal amounts of cells (lysates) or volumes (supernatants) were analyzed by WB. Compare the significantly higher amounts of Shh detected in cell lysates of Shh-transfected HeLa cells. (B) Density of Shh and apolipoproteins in MIA PaCa-2 cell supernatants shown in (A), analyzed by Optiprep density gradient centrifugation. Gradients were analyzed by WB to detect Shh (see also Figure 1G), and by Commassie staining of gels to detect apolipoproteins. (C) Density of Hh in supernatants from S2 cells expressing *Drosophila* Hh in serum-free medium supplemented with Lpp, analyzed by Optiprep density gradient centrifugation and WB. (D) Density of Hh in supernatants of S2 cells expressing *Drosophila* Hh grown in medium containing 10% FCS, analyzed by KBr density gradient centrifugation and WB.
(TIF)

Figure S3 Properties of circulating *Drosophila* Hh and human Shh. (A) Shh is present in lipoprotein-containing fractions in human circulation. Lipoproteins were isolated from 4 ml of human serum (Sigma) by KBr density centrifugation [65]. Membranous vesicles (along with large lipoproteins such as chylomicrons and VLDL—see also Figure S12A) were pelleted by centrifugation at 100,000 *g* for 2 h, and resulting supernatants subsequently analyzed by Optiprep density gradient centrifugation and WB. Colors indicate fractions corresponding to human Very Low-, Low-, and High-Density Lipoproteins (VLDL, LDL, and HDL) [43]. (B) Hemolymph Hh levels in larvae expressing Hh RNAi in imaginal discs (*hh*-GAL4) or ubiquitously (*tubulin*-GAL4). Hh knock-down in imaginal discs does not reduce the levels of hemolymph Hh. In contrast ubiquitous knock-down strongly depletes Hh from the hemolymph. (C) *Hh* RT-PCR on cDNA prepared from total RNA extracts from larval fat body (without gonads) and wing discs. Actin was used as a positive control. The actin primers were designed to span an intron to allow detection of possible contamination of cDNA preparations with genomic DNA. Note that Hh transcripts can be detected in the wing disc, but not in the larval fat body. (D) Density of hemolymph Hh and lipoproteins, analyzed by KBr density gradient centrifugation and WB. Note that *Drosophila* lipoproteins are separated more completely in these KBr gradients than in Optiprep gradients (compare Figure S3G). (E) Density of hemolymph Hh from normally fed or lipid-starved larvae, analyzed by Optiprep density gradient centrifugation and WB. Note that lipid-starvation increases the density of Lpp [26]. (F) Hydrophobicity of hemolymph Hh from normally fed or lipid-starved larvae, analyzed by Triton X-114 phase separation and WB. Note that removal of lipids (including sterols) from the diet does not alter Hh hydrophobicity. (G) Density of hemolymph Hh of wild-type and Lpp RNAi larvae, analyzed by Optiprep density gradient centrifugation and WB. Lpp RNAi causes the appearance of a population of hemolymph Hh that has a higher density than any *Drosophila* lipoprotein. Note that Lpp is by far the most abundant lipoprotein in *Drosophila* larvae [26].
(TIF)

Figure S4 The *Drosophila* hemolymph as a system to study Hh secretion. (A) Hemolymph Hh levels in larvae expressing Hh under the control of different GAL4 drivers, analyzed by WB. *lpp*-GAL4 is strongly active in the fat body; *myoIA*-GAL4 is mostly and strongly active in the gut; *npc1b*-GAL4 is moderately active in the midgut; *en105*-GAL4 is mostly and strongly active in the posterior compartment of imaginal discs. Cv-d is used as a loading control. (B) Hh levels in hemolymph and fat body of control and Dispatched (Disp) mutant larvae ectopically expressing Hh in the fat body, analyzed by WB. Note that hemolymph levels of fat-body-secreted Hh are strongly decreased in Dispatched mutants. Loading controls are Cv-d (hemolymph) or tubulin (larval extract). (C) Hh levels in hemolymph and gut of control and Dispatched mutant larvae ectopically expressing Hh in the midgut. Note that hemolymph levels of midgut-secreted Hh are strongly decreased in Dispatched mutants. Loading controls are Cv-d (hemolymph) or tubulin (larval extract). (D) Density of hemolymph Hh in larvae expressing Hh under the control of *lpp*-GAL4 (see also Figure 3B), *npc1b*-GAL4, or *en105*-GAL4 (see also Figure S1E), analyzed by Optiprep density gradient centrifugation and WB. Note that different amounts of hemolymph were analyzed for the different GAL4 lines; for absolute levels of hemolymph Hh under these conditions, see (A). (E) WB of Hh secretion time course. Hh was expressed in the fat body in a time-controlled manner using *lpp*-GAL4, *tubulin*-GAL80^TS^. Hemolymph was collected after the indicated periods of time, and equal amounts fractionated in Optiprep density gradients. (F) Immunofluorescence of fat body and wing disc from larvae expressing UAS-CD8-GFP with *lpp*-GAL4. Membranes are stained with FM-4-6-4. GFP can be readily detected in the fat body, but not in the wing disc. Scale bar = 50 µm. (G) Hemolymph Hh levels in larvae expressing Hh lipid modification variants in the fat body. Note that ectopic of any Hh variant strongly elevates hemolymph Hh levels compared to wild type. Loading control is apoLTPII.
(TIF)

Figure S5 Molecular properties of different Hh/Shh secretion forms. (A) WB of Shh fractionated by size and density. Supernatants from Shh-transfected HeLa cells grown in the presence of FBS were analyzed by gel filtration chromatography. Column fractions were pooled as indicated and subsequently analyzed by both Triton-X 114 phase separation and Optiprep density gradient centrifugation. (B) WB of hemolymph Hh fractionated by size and density. Fat-body-secreted hemolymph Hh was analyzed by gel filtration chromatography. Column fractions were pooled as indicated, and subsequently analyzed by Optiprep density gradient centrifugation. (C) Quantification of the elution profiles of hemolymph Hh secreted from wild-type and Lpp RNAi fat bodies (see Figure 3D). Hh band intensity in each fraction is depicted as percentage of the combined Hh signal of all column fractions. (D) Hydrophobicity of Shh lipid modification mutants. Different Shh mutants were expressed in HeLa cells grown in serum-containing medium, and resulting supernatants analyzed by Triton X-114 phase separation and WB. (E) Hydrophobicity of Hh lipid modification mutants. Different Hh variants were secreted to the hemolymph from the fat body, and their hydrophobicity assessed by Triton X-114 phase separation. Sterol-modified Hh/Shh and Hh^C85S^/Shh^C24S^ partition predominantly into the detergent (det) phase. Hh variants lacking sterol modification (Hh-N/Shh-N and Hh-N^C85S^/Shh-N^C24S^) partition predominantly into the aqueous phase (aq). (F) Hydrophobicity and electrophoretic mobility of Hh, Hh-N*, and Hh-N^C85S^, assessed by Triton X-114 phase separation and WB. Note the similar electrophoretic mobility of aqueous phase Hh-N* and Hh-N^C85S^. See also Figure 3H,I.
(TIF)

Figure S6 Lipoprotein-independent Hh secretion forms in imaginal discs. (A and B) Quantification of (A) Ci_155_ and (B) Engrailed staining of wing discs from larvae in which Lpp, LTP, or Cv-d was knocked down in the fat body by RNAi. Lpp RNAi stabilizes Ci_155_ throughout the anterior compartment (see also Figure 5B,D) and reduces the range of Engrailed expression close to the compartment boundary. LTP or Cv-d RNAi does not detectably affect Hh signaling. Yellow lines indicate the anterior/posterior compartment boundary. Translucent lines indicate ±SD (*n* = 10). (C) Hemolymph from larvae secreting Hh from the fat body was diluted 1∶10 with PBS, 100 mM Na_2_CO_3_ pH 9, or 100 mM Na_2_CO_3_ pH 10 and incubated for 24 h. Subsequently, Hh hydrophobicity was analyzed by Triton X-114 phase separation and WB. pH 9 or pH 10 does not increase the levels of Hh present in the aqueous phase, indicating that these conditions do not hydrolyze the ester bond between Hh and sterol. (D) Effect of mildly alkaline pH or high salt on the recovery of high-density Hh from *Drosophila* imaginal discs. The 100,000 *g* supernatants were subjected to KBr density gradient centrifugation and gradient fractions analyzed by WB. The same number of everted heads was processed for each gradient. Note that high-density Hh is completely undetectable in 100,000 *g* supernatants of imaginal discs dissociated in PBS.
(TIF)

Figure S7 *Drosophila* embryos produce Hh-N*. (A) Experimental scheme to purify Hh-N* from *Drosophila* embryos. Embryonic extracts for experiments shown in (B–F) were prepared with 100 mM Na_2_CO_3_, pH 9. Embryonic extracts for experiments shown in (H) and (I) were prepared in 10 mM Tris-HCl, pH 8, 0.5 M NaCl. All buffers used for preparation of embryonic extracts contained 0.05% NP-40. (B) Recovery of Hh in 16,000 *g* (S1) and 100,000 *g* (S2) supernatants from embryonic extracts, analyzed by WB. Equivalent amounts of S1 and S2 were loaded. (C) Density of soluble Hh in S2 from embryonic extracts, analyzed by KBr density gradient centrifugation and WB. (D) Hydrophobicity of low-density and high-density Hh from (C), assessed by Triton X-114 phase separation. (E) Size of low-density and high-density Hh from (C), assessed by gel filtration chromatography and WB. (F) Hydrophobicity of high-density/low-molecular-weight Hh from (E), assessed by Triton X-114 phase separation. (G) Effect of high salt on the recovery of high-density Hh from *Drosophila* embryos. The 100,000 *g* supernatants were fractionated in KBr density gradients and analyzed by WB. Similar volumes of embryos were processed for each gradient. Gradients showing extracts prepared with PBS ±0.5 M NaCl were analyzed in the same experiment; extracts prepared with 10 mM Tris-HCl, pH 8, 0.5 M NaCl were processed and analyzed separately. (H) Hydrophobicity of low-density and high-density Hh recovered by high salt conditions shown in (G), assessed by Triton X-114 phase separation and WB. (I) Size of high-density Hh recovered by high salt conditions, assessed by gel filtration chromatography and WB.
(TIF)

Figure S8 Effects of hemolymph Lpp-associated Hh and Lpp RNAi on Hh signaling in the wing imaginal disc. (A) Wing discs from larvae secreting moderate levels of Hh into the hemolymph under the control of *npc1b*-GAL4 or high levels under the control of *lpp*-GAL4, stained for Hh and Ci_155_ (see also Figure S3A). *npc1b*-GAL4 does not drive sufficient Hh expression to influence signaling in the disc. We expect therefore that the much lower endogenous levels of circulating Hh are insufficient to influence patterning in discs. Furthermore, *en105*-GAL4 produces even less circulating Hh than *npc1b*-GAL4 (see Figure 4A). Thus, the small increase in circulating Hh caused by *en105*-GAL4–driven Hh expression is unlikely to contribute to increased Hh signaling in *en105*-GAL4>UAS Hh wing discs. Scale bar = 50 µm. (B and C) Quantification of (B) Hh, (C) Ci_155_, staining of wing discs shown in (A). Yellow lines indicate the anterior/posterior compartment boundary. Translucent lines indicate ±SD (*n* = 10). (D) Quantification of Smoothened staining in wing discs from larvae secreting Hh from the fat body. Note that Lpp-associated Hh secreted from the fat body increases Smoothened levels in the anterior compartment. Yellow lines indicate the anterior/posterior compartment boundary. Translucent lines indicate ±SD (*n* = 10). (E) Quantification of Ci_75_ levels in wing discs from larvae expressing Hh or Lpp RNAi in the fat body. Band intensity of Western blots was quantified and normalized to α-tubulin. Error bars indicate ±SD (*n* = 5). (F) Quantification of Engrailed in wing discs from Lpp RNAi larvae or larvae secreting Hh from the fat body. Note that expression of the high-threshold Hh target gene Engrailed is strongly repressed in the wing disc by high levels of Lpp-associated Hh. Yellow lines indicate the anterior/posterior compartment boundary. Translucent lines indicate ±SD (*n* = 12). (G) Immunofluorescence of wing discs from Lpp RNAi larvae or larvae secreting Hh from the fat body, stained for Hh, Ci_155_, and Engrailed. Scale bar = 20 µm.
(TIF)

Figure S9 Signaling properties of Hh-N*. (A) Immunofluorescence of wing discs from larvae secreting Hh or Hh-N* from the fat body, stained for Hh and Ci_155_. Hh-N* was generated by expressing Hh in the fat body of Lpp RNAi animals. Scale bar = 100 µm. (B) Quantification of Hh staining of wing discs shown in (A). Yellow lines indicate the anterior/posterior compartment boundary. Translucent lines indicate ±SD (*n* = 10).
(TIF)

Figure S10 Signaling properties of Hh-N. (A) Immunofluorescence of wing discs from larvae secreting Hh or Hh-N^Med^ from the fat body, stained for Hh, Ci_155_, and Engrailed. Scale bar = 50 µm. (B–D) Quantification of (B) Hh, (C) Ci_155_, and (D) Engrailed staining of wing discs shown in (A). Translucent lines indicate ±SD (*n* = 12). (E) Quantification of Ci_75_ levels in wing discs from larvae expressing Hh or Hh-N^Med^ in the fat body. Band intensity of Western blots was quantified and normalized to α-tubulin. Error bars indicate ±SD (*n* = 3); *p<0.05. (F) Quantification of Ci_75_ levels in wing discs from larvae expressing Hh and Hh-N^Low^, alone or in combination, in the fat body. Band intensity of Western blots was quantified and normalized to α-tubulin. Error bars indicate ±SD (*n* = 5); *p<0.05. (G) Immunofluorescence of wing discs from larvae secreting Hh and Hh-N^Low^, alone or in combination, from the fat body, stained for Hh, Ci_155_, and *dpp*LacZ. Scale bar = 50 µm.
(TIF)

Figure S11 Lipoproteins repress the signaling activity of Shh-N*. (A) Shh-N* isolated from HeLa cells grown in serum-free medium was incubated in PBS containing 1% FBS at 37°C with constant shaking and subsequently fractionated by density gradient centrifugation. Shh-N* that was not exposed to serum served as a control. After incubation with serum, the vast majority of Shh-N* was still present in fractions of the highest density, suggesting that it failed to associate with lipoproteins. (B) Concentration-dependent repression of signaling activity of Shh-N* by lipoproteins in Shh-LIGHT2 cells. The concentration of lipoprotein-associated Shh or Shh-N* was kept constant, and only the amount of lipoproteins increased. Note that the starting amount of lipoproteins in the case of lipoprotein-associated Shh already corresponds to 10 arbitrary units. Error bars indicate ±SD (*n* = 3; *p<0.05, **p<0.005, ***p<0.0005). Shown is one representative experiment out of three.
(TIF)

Figure S12 Ultracentrifuge sedimentation and Triton X-114 phase separation behavior of lipoproteins and Hh proteins. (A) Different human lipoprotein classes isolated from human serum were centrifuged at 100,000 *g* for 2 h. Equal volumes of input and 100,000 *g* supernatants (S) were separated by gel electrophoresis and visualized by Coomassie staining. Note that centrifugation at 100,000 *g* completely pellets VLDL and partially pellets LDL particles. HDL levels in 100,000 *g* supernatants are not significantly affected compared to input samples. (B) *Drosophila* larval hemolymph was centrifuged at the indicated speeds; resulting pellets and supernatants were analyzed by WB. The Lpp scaffolding protein apoLI was visualized by Ponceau S staining of the nitrocellulose membrane, the LTP scaffolding protein apoLTPII by immunodetection. Note that all detectable LTP and a large fraction of the smaller Lpp are pelleted by centrifugation at 100,000 *g*. (C) Lipoproteins isolated from FBS were subjected to Triton X-114 phase separation, alone or after mixing with Shh-N*. Note that both the HDL scaffolding apolipoprotein apoA1 and Shh-N* partition into the aqueous phase. Similarly, Shh-N* partitions into the aqueous phase in the absence of lipoproteins (see Figure 3G).
(TIF)

## Abbreviations

apoA1: apolipoprotein A1
apoB: apolipoprotein B
apoLI: apoLipophorin I
apoLII: apoLipophorin II
Ci: cubitus interruptus
Col: collier
Cv-d: crossveinless-d
Disp: dispatched
Dpp: decapentaplegic
En: engrailed
HDL: high-density lipoprotein
Hh: hedgehog
LDL: low-density lipoprotein
Lpp: lipophorin
LTP: lipid transfer particle
MTP: microsomal triglyceride transfer protein
Ptc: patched
Shh: sonic hedgehog
Smo: smoothened
VLDL: very low-density lipoprotein
