# Supplementary figures and images for: Secretion and Signaling Activities of Lipoprotein-Associated Hedgehog and Non-Sterol-Modified Hedgehog in Flies and Mammals
Source: PLoS Biol. 2013 Mar 12;11(3):e1001505. doi: 10.1371/journal.pbio.1001505 (PMC3595218; doi:10.1371/journal.pbio.1001505)

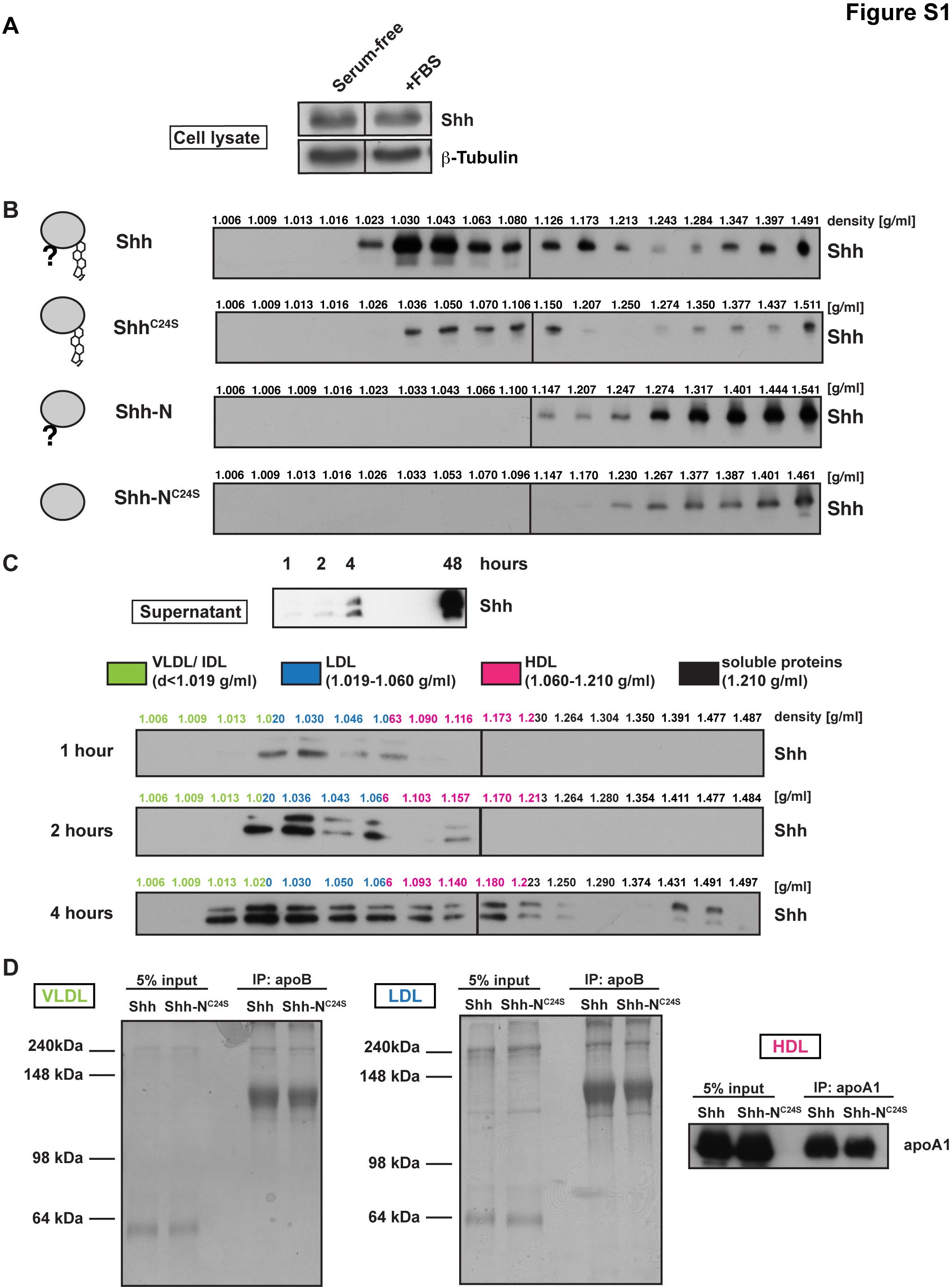

Supplement: Figure S1 — Shh is secreted in lipoprotein-associated and lipoprotein-free forms. (A) Shh levels in cell lysates from HeLa cells transfected with human Shh, grown in serum-free medium or in the presence of 10% FBS, analyzed by Western blotting (WB). (B) Density of different Shh lipid modification mutants. Supernatants from HeLa cells transfected with Shh, ShhC24S, Shh-N, or Shh-NC24S and grown in the presence of FBS were analyzed by Optiprep density gradient centrifugation and WB. Note that it is difficult to determine whether palmitate might suffice for lipoprotein association, since mammalian tissue culture cells overexpressing Shh do not palmitoylate it efficiently [5],[69]. (C) WB of Shh secretion time course. HeLa cells were transfected with Shh and grown in serum-free medium. 24 h after transfection, medium was replaced by fresh medium supplemented with 10% FBS; supernatants were collected after the indicated periods of time, and equal volumes analyzed by Optiprep density gradient centrifugation. Colors indicate fractions corresponding to bovine Very Low-, Low-, and High-Density Lipoproteins (VLDL, LDL, and HDL) [68]. (D) Immunoprecipitation of different human lipoprotein classes from HeLa cell supernatants. ApoB was detected by Coomassie staining of gels; apoA1 was detected by WB. The same samples were analyzed for co-immunoprecipitated Shh in Figure 1E. (TIF) [file pbio.1001505.s001.tif]

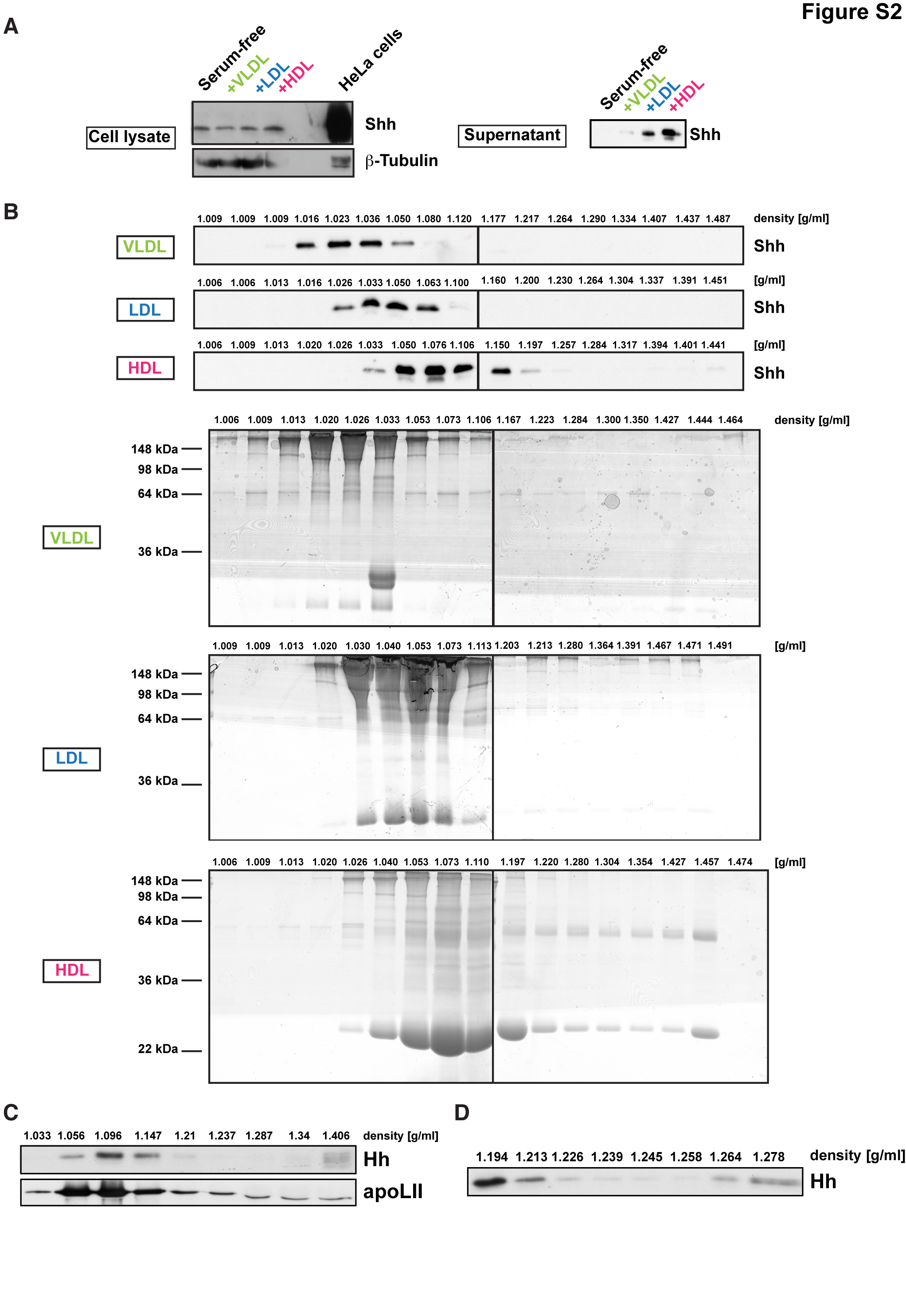

Supplement: Figure S2 — Hh proteins can associate with different human and Drosophila lipoproteins. (A) Shh levels in supernatants and cell lysates derived from MIA PaCa-2, grown in serum-free medium with or without addition of different human lipoproteins. Equal amounts of cells (lysates) or volumes (supernatants) were analyzed by WB. Compare the significantly higher amounts of Shh detected in cell lysates of Shh-transfected HeLa cells. (B) Density of Shh and apolipoproteins in MIA PaCa-2 cell supernatants shown in (A), analyzed by Optiprep density gradient centrifugation. Gradients were analyzed by WB to detect Shh (see also Figure 1G), and by Commassie staining of gels to detect apolipoproteins. (C) Density of Hh in supernatants from S2 cells expressing Drosophila Hh in serum-free medium supplemented with Lpp, analyzed by Optiprep density gradient centrifugation and WB. (D) Density of Hh in supernatants of S2 cells expressing Drosophila Hh grown in medium containing 10% FCS, analyzed by KBr density gradient centrifugation and WB. (TIF) [file pbio.1001505.s002.tif]

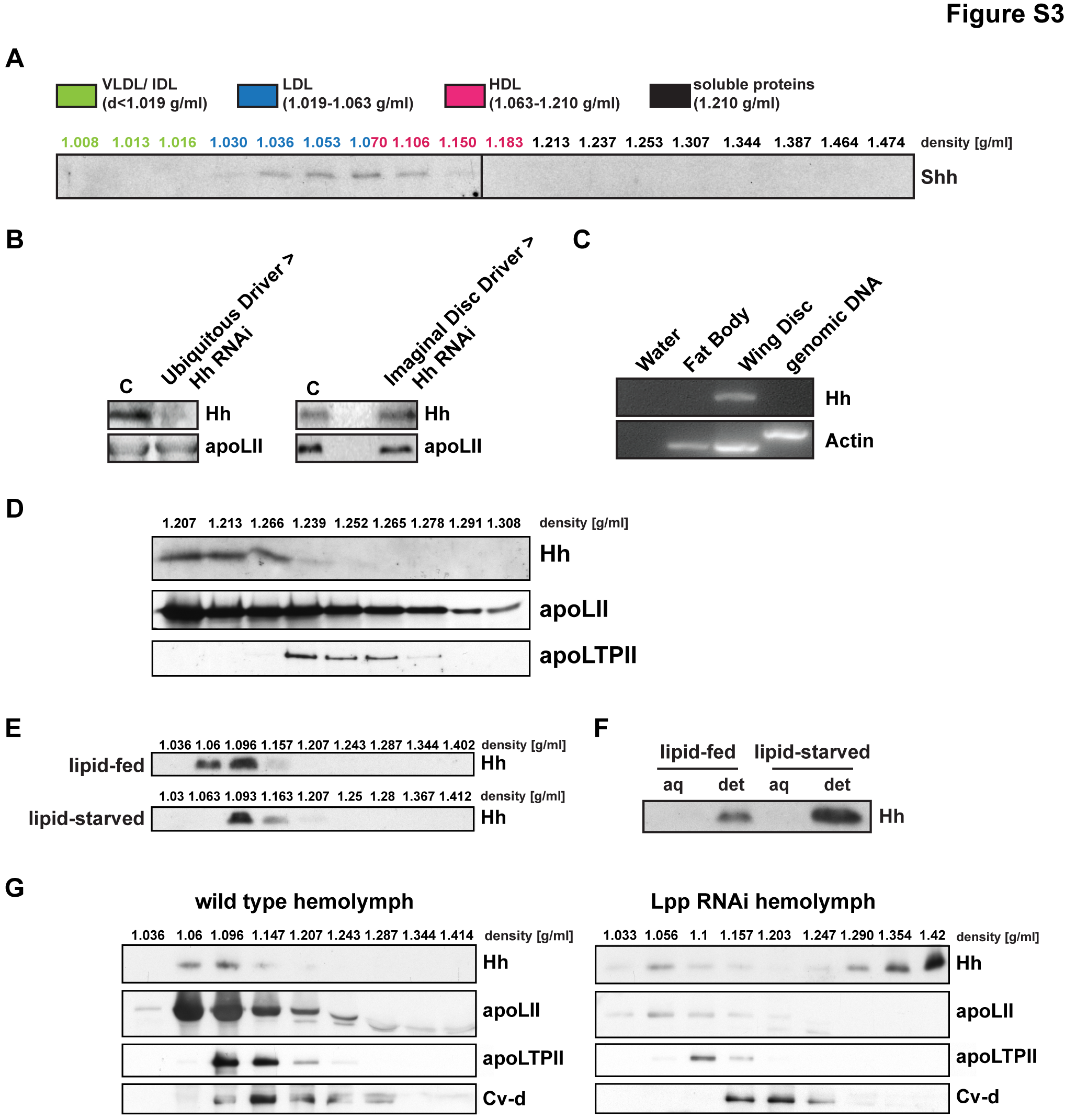

Supplement: Figure S3 — Properties of circulating Drosophila Hh and human Shh. (A) Shh is present in lipoprotein-containing fractions in human circulation. Lipoproteins were isolated from 4 ml of human serum (Sigma) by KBr density centrifugation [65]. Membranous vesicles (along with large lipoproteins such as chylomicrons and VLDL—see also Figure S12A) were pelleted by centrifugation at 100,000 g for 2 h, and resulting supernatants subsequently analyzed by Optiprep density gradient centrifugation and WB. Colors indicate fractions corresponding to human Very Low-, Low-, and High-Density Lipoproteins (VLDL, LDL, and HDL) [43]. (B) Hemolymph Hh levels in larvae expressing Hh RNAi in imaginal discs (hh-GAL4) or ubiquitously (tubulin-GAL4). Hh knock-down in imaginal discs does not reduce the levels of hemolymph Hh. In contrast ubiquitous knock-down strongly depletes Hh from the hemolymph. (C) Hh RT-PCR on cDNA prepared from total RNA extracts from larval fat body (without gonads) and wing discs. Actin was used as a positive control. The actin primers were designed to span an intron to allow detection of possible contamination of cDNA preparations with genomic DNA. Note that Hh transcripts can be detected in the wing disc, but not in the larval fat body. (D) Density of hemolymph Hh and lipoproteins, analyzed by KBr density gradient centrifugation and WB. Note that Drosophila lipoproteins are separated more completely in these KBr gradients than in Optiprep gradients (compare Figure S3G). (E) Density of hemolymph Hh from normally fed or lipid-starved larvae, analyzed by Optiprep density gradient centrifugation and WB. Note that lipid-starvation increases the density of Lpp [26]. (F) Hydrophobicity of hemolymph Hh from normally fed or lipid-starved larvae, analyzed by Triton X-114 phase separation and WB. Note that removal of lipids (including sterols) from the diet does not alter Hh hydrophobicity. (G) Density of hemolymph Hh of wild-type and Lpp RNAi larvae, analyzed by Optiprep density gradient [file pbio.1001505.s003.tif]

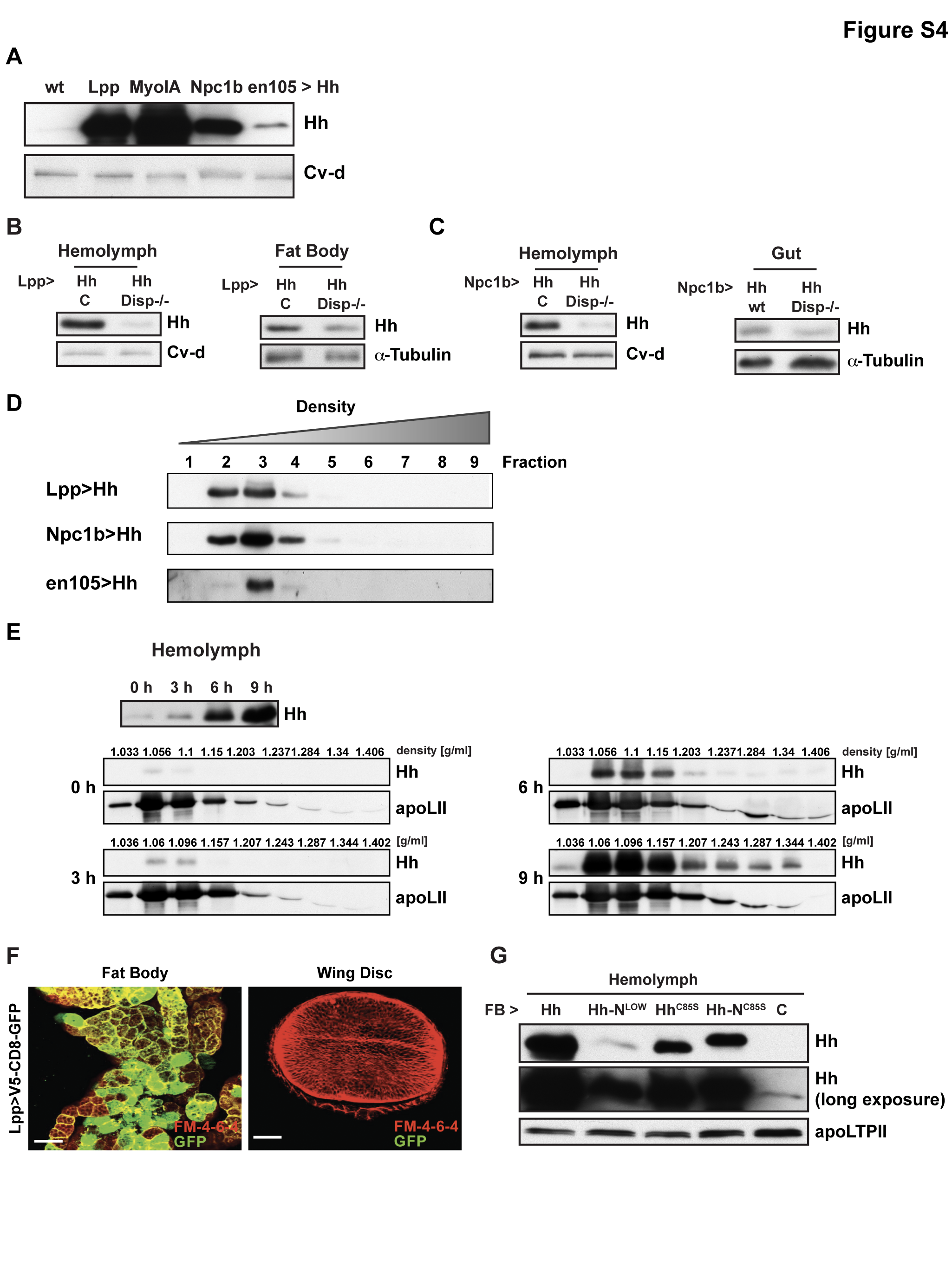

Supplement: Figure S4 — The Drosophila hemolymph as a system to study Hh secretion. (A) Hemolymph Hh levels in larvae expressing Hh under the control of different GAL4 drivers, analyzed by WB. lpp-GAL4 is strongly active in the fat body; myoIA-GAL4 is mostly and strongly active in the gut; npc1b-GAL4 is moderately active in the midgut; en105-GAL4 is mostly and strongly active in the posterior compartment of imaginal discs. Cv-d is used as a loading control. (B) Hh levels in hemolymph and fat body of control and Dispatched (Disp) mutant larvae ectopically expressing Hh in the fat body, analyzed by WB. Note that hemolymph levels of fat-body-secreted Hh are strongly decreased in Dispatched mutants. Loading controls are Cv-d (hemolymph) or tubulin (larval extract). (C) Hh levels in hemolymph and gut of control and Dispatched mutant larvae ectopically expressing Hh in the midgut. Note that hemolymph levels of midgut-secreted Hh are strongly decreased in Dispatched mutants. Loading controls are Cv-d (hemolymph) or tubulin (larval extract). (D) Density of hemolymph Hh in larvae expressing Hh under the control of lpp-GAL4 (see also Figure 3B), npc1b-GAL4, or en105-GAL4 (see also Figure S1E), analyzed by Optiprep density gradient centrifugation and WB. Note that different amounts of hemolymph were analyzed for the different GAL4 lines; for absolute levels of hemolymph Hh under these conditions, see (A). (E) WB of Hh secretion time course. Hh was expressed in the fat body in a time-controlled manner using lpp-GAL4, tubulin-GAL80TS. Hemolymph was collected after the indicated periods of time, and equal amounts fractionated in Optiprep density gradients. (F) Immunofluorescence of fat body and wing disc from larvae expressing UAS-CD8-GFP with lpp-GAL4. Membranes are stained with FM-4-6-4. GFP can be readily detected in the fat body, but not in the wing disc. Scale bar = 50 µm. (G) Hemolymph Hh levels in larvae expressing Hh lipid modification variants in the fat body. Note that ectopic of any Hh varian [file pbio.1001505.s004.tif]

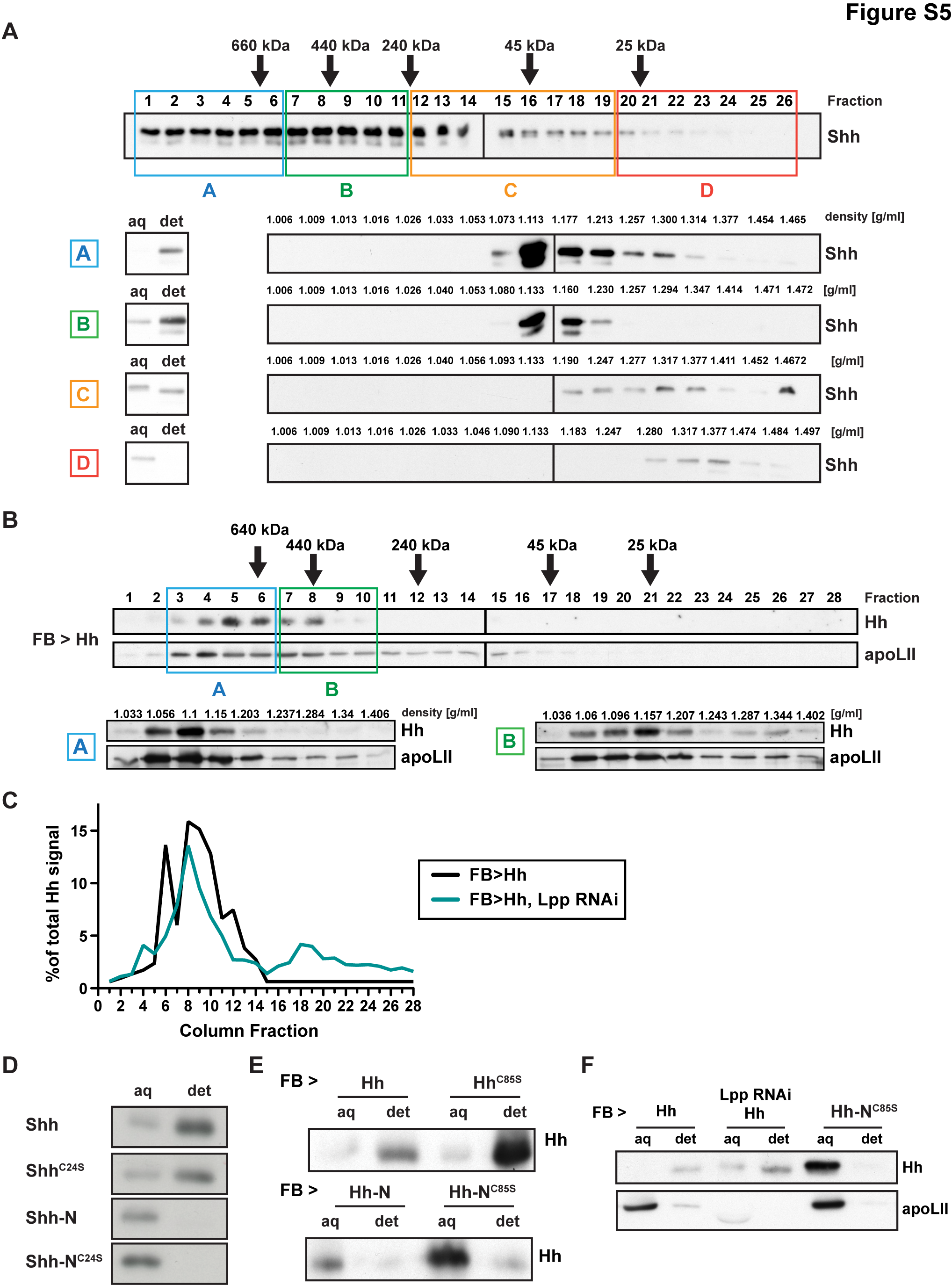

Supplement: Figure S5 — Molecular properties of different Hh/Shh secretion forms. (A) WB of Shh fractionated by size and density. Supernatants from Shh-transfected HeLa cells grown in the presence of FBS were analyzed by gel filtration chromatography. Column fractions were pooled as indicated and subsequently analyzed by both Triton-X 114 phase separation and Optiprep density gradient centrifugation. (B) WB of hemolymph Hh fractionated by size and density. Fat-body-secreted hemolymph Hh was analyzed by gel filtration chromatography. Column fractions were pooled as indicated, and subsequently analyzed by Optiprep density gradient centrifugation. (C) Quantification of the elution profiles of hemolymph Hh secreted from wild-type and Lpp RNAi fat bodies (see Figure 3D). Hh band intensity in each fraction is depicted as percentage of the combined Hh signal of all column fractions. (D) Hydrophobicity of Shh lipid modification mutants. Different Shh mutants were expressed in HeLa cells grown in serum-containing medium, and resulting supernatants analyzed by Triton X-114 phase separation and WB. (E) Hydrophobicity of Hh lipid modification mutants. Different Hh variants were secreted to the hemolymph from the fat body, and their hydrophobicity assessed by Triton X-114 phase separation. Sterol-modified Hh/Shh and HhC85S/ShhC24S partition predominantly into the detergent (det) phase. Hh variants lacking sterol modification (Hh-N/Shh-N and Hh-NC85S/Shh-NC24S) partition predominantly into the aqueous phase (aq). (F) Hydrophobicity and electrophoretic mobility of Hh, Hh-N*, and Hh-NC85S, assessed by Triton X-114 phase separation and WB. Note the similar electrophoretic mobility of aqueous phase Hh-N* and Hh-NC85S. See also Figure 3H,I. (TIF) [file pbio.1001505.s005.tif]

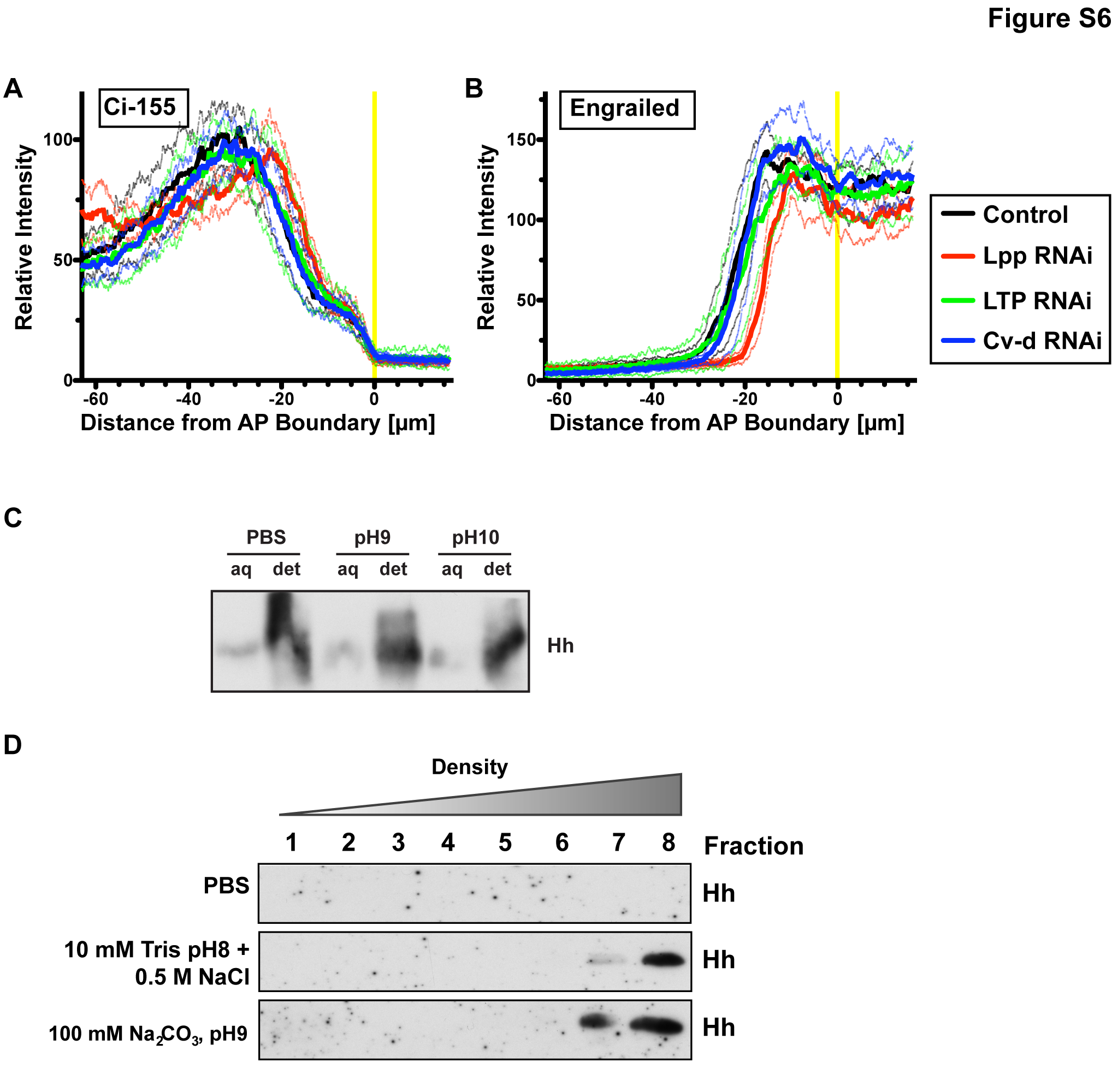

Supplement: Figure S6 — Lipoprotein-independent Hh secretion forms in imaginal discs. (A and B) Quantification of (A) Ci155 and (B) Engrailed staining of wing discs from larvae in which Lpp, LTP, or Cv-d was knocked down in the fat body by RNAi. Lpp RNAi stabilizes Ci155 throughout the anterior compartment (see also Figure 5B,D) and reduces the range of Engrailed expression close to the compartment boundary. LTP or Cv-d RNAi does not detectably affect Hh signaling. Yellow lines indicate the anterior/posterior compartment boundary. Translucent lines indicate ±SD (n = 10). (C) Hemolymph from larvae secreting Hh from the fat body was diluted 1∶10 with PBS, 100 mM Na2CO3 pH 9, or 100 mM Na2CO3 pH 10 and incubated for 24 h. Subsequently, Hh hydrophobicity was analyzed by Triton X-114 phase separation and WB. pH 9 or pH 10 does not increase the levels of Hh present in the aqueous phase, indicating that these conditions do not hydrolyze the ester bond between Hh and sterol. (D) Effect of mildly alkaline pH or high salt on the recovery of high-density Hh from Drosophila imaginal discs. The 100,000 g supernatants were subjected to KBr density gradient centrifugation and gradient fractions analyzed by WB. The same number of everted heads was processed for each gradient. Note that high-density Hh is completely undetectable in 100,000 g supernatants of imaginal discs dissociated in PBS. (TIF) [file pbio.1001505.s006.tif]

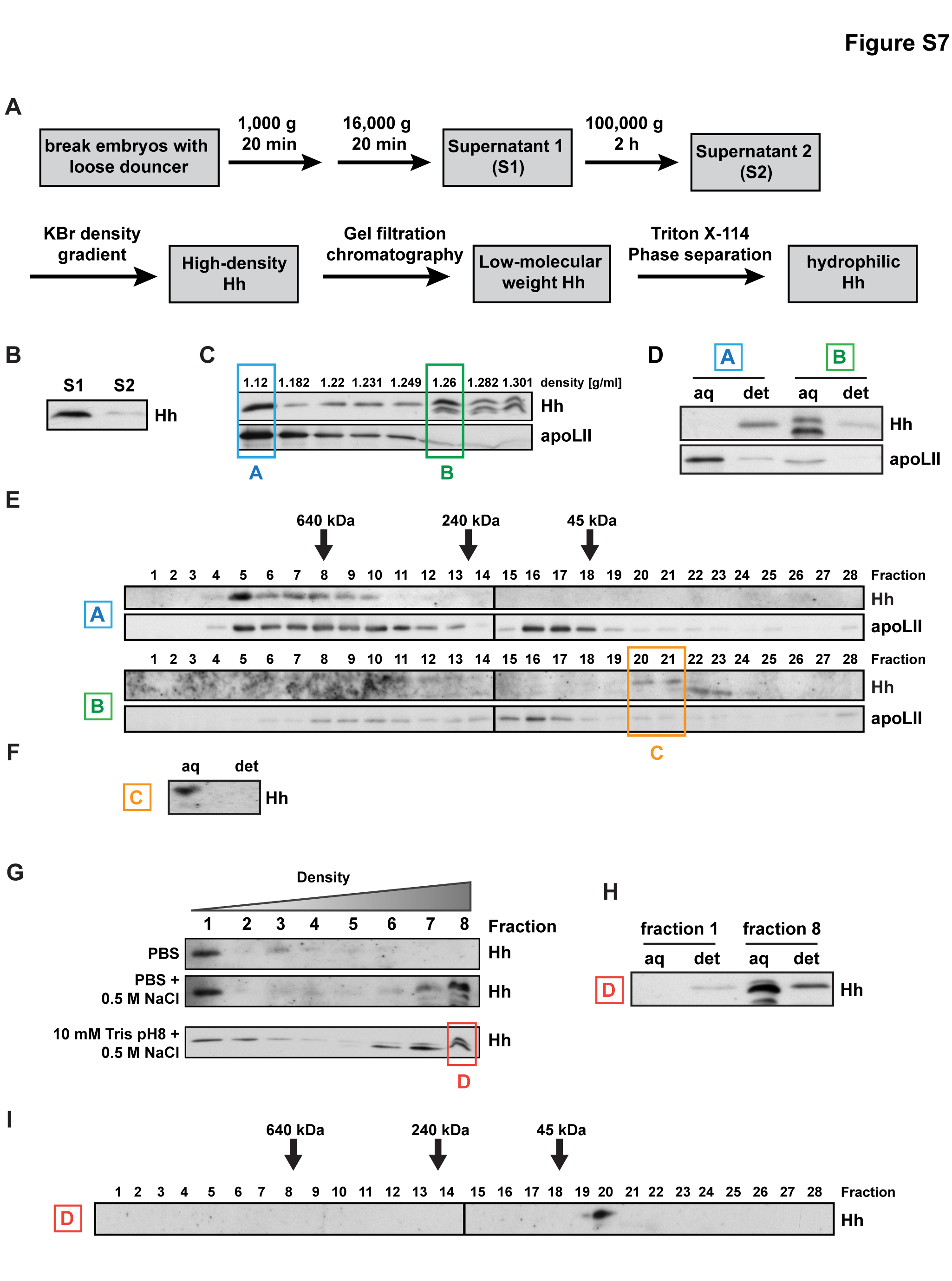

Supplement: Figure S7 — Drosophila embryos produce Hh-N*. (A) Experimental scheme to purify Hh-N* from Drosophila embryos. Embryonic extracts for experiments shown in (B–F) were prepared with 100 mM Na2CO3, pH 9. Embryonic extracts for experiments shown in (H) and (I) were prepared in 10 mM Tris-HCl, pH 8, 0.5 M NaCl. All buffers used for preparation of embryonic extracts contained 0.05% NP-40. (B) Recovery of Hh in 16,000 g (S1) and 100,000 g (S2) supernatants from embryonic extracts, analyzed by WB. Equivalent amounts of S1 and S2 were loaded. (C) Density of soluble Hh in S2 from embryonic extracts, analyzed by KBr density gradient centrifugation and WB. (D) Hydrophobicity of low-density and high-density Hh from (C), assessed by Triton X-114 phase separation. (E) Size of low-density and high-density Hh from (C), assessed by gel filtration chromatography and WB. (F) Hydrophobicity of high-density/low-molecular-weight Hh from (E), assessed by Triton X-114 phase separation. (G) Effect of high salt on the recovery of high-density Hh from Drosophila embryos. The 100,000 g supernatants were fractionated in KBr density gradients and analyzed by WB. Similar volumes of embryos were processed for each gradient. Gradients showing extracts prepared with PBS ±0.5 M NaCl were analyzed in the same experiment; extracts prepared with 10 mM Tris-HCl, pH 8, 0.5 M NaCl were processed and analyzed separately. (H) Hydrophobicity of low-density and high-density Hh recovered by high salt conditions shown in (G), assessed by Triton X-114 phase separation and WB. (I) Size of high-density Hh recovered by high salt conditions, assessed by gel filtration chromatography and WB. (TIF) [file pbio.1001505.s007.tif]

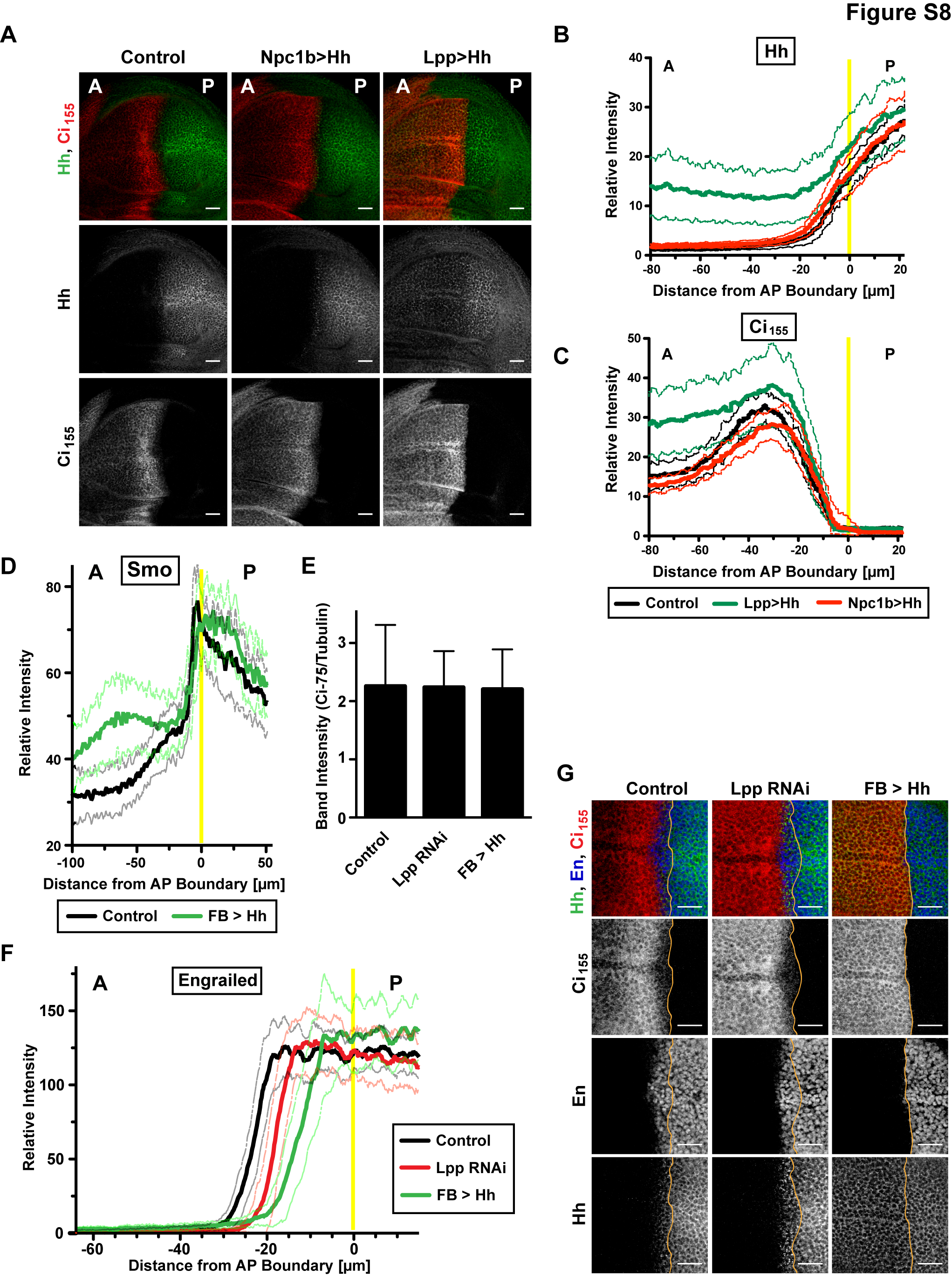

Supplement: Figure S8 — Effects of hemolymph Lpp-associated Hh and Lpp RNAi on Hh signaling in the wing imaginal disc. (A) Wing discs from larvae secreting moderate levels of Hh into the hemolymph under the control of npc1b-GAL4 or high levels under the control of lpp-GAL4, stained for Hh and Ci155 (see also Figure S3A). npc1b-GAL4 does not drive sufficient Hh expression to influence signaling in the disc. We expect therefore that the much lower endogenous levels of circulating Hh are insufficient to influence patterning in discs. Furthermore, en105-GAL4 produces even less circulating Hh than npc1b-GAL4 (see Figure 4A). Thus, the small increase in circulating Hh caused by en105-GAL4–driven Hh expression is unlikely to contribute to increased Hh signaling in en105-GAL4>UAS Hh wing discs. Scale bar = 50 µm. (B and C) Quantification of (B) Hh, (C) Ci155, staining of wing discs shown in (A). Yellow lines indicate the anterior/posterior compartment boundary. Translucent lines indicate ±SD (n = 10). (D) Quantification of Smoothened staining in wing discs from larvae secreting Hh from the fat body. Note that Lpp-associated Hh secreted from the fat body increases Smoothened levels in the anterior compartment. Yellow lines indicate the anterior/posterior compartment boundary. Translucent lines indicate ±SD (n = 10). (E) Quantification of Ci75 levels in wing discs from larvae expressing Hh or Lpp RNAi in the fat body. Band intensity of Western blots was quantified and normalized to α-tubulin. Error bars indicate ±SD (n = 5). (F) Quantification of Engrailed in wing discs from Lpp RNAi larvae or larvae secreting Hh from the fat body. Note that expression of the high-threshold Hh target gene Engrailed is strongly repressed in the wing disc by high levels of Lpp-associated Hh. Yellow lines indicate the anterior/posterior compartment boundary. Translucent lines indicate ±SD (n = 12). (G) Immunofluorescence of wing discs from Lpp RNAi larvae or larvae secreting Hh from the fat body, stained for Hh, Ci155, [file pbio.1001505.s008.tif]

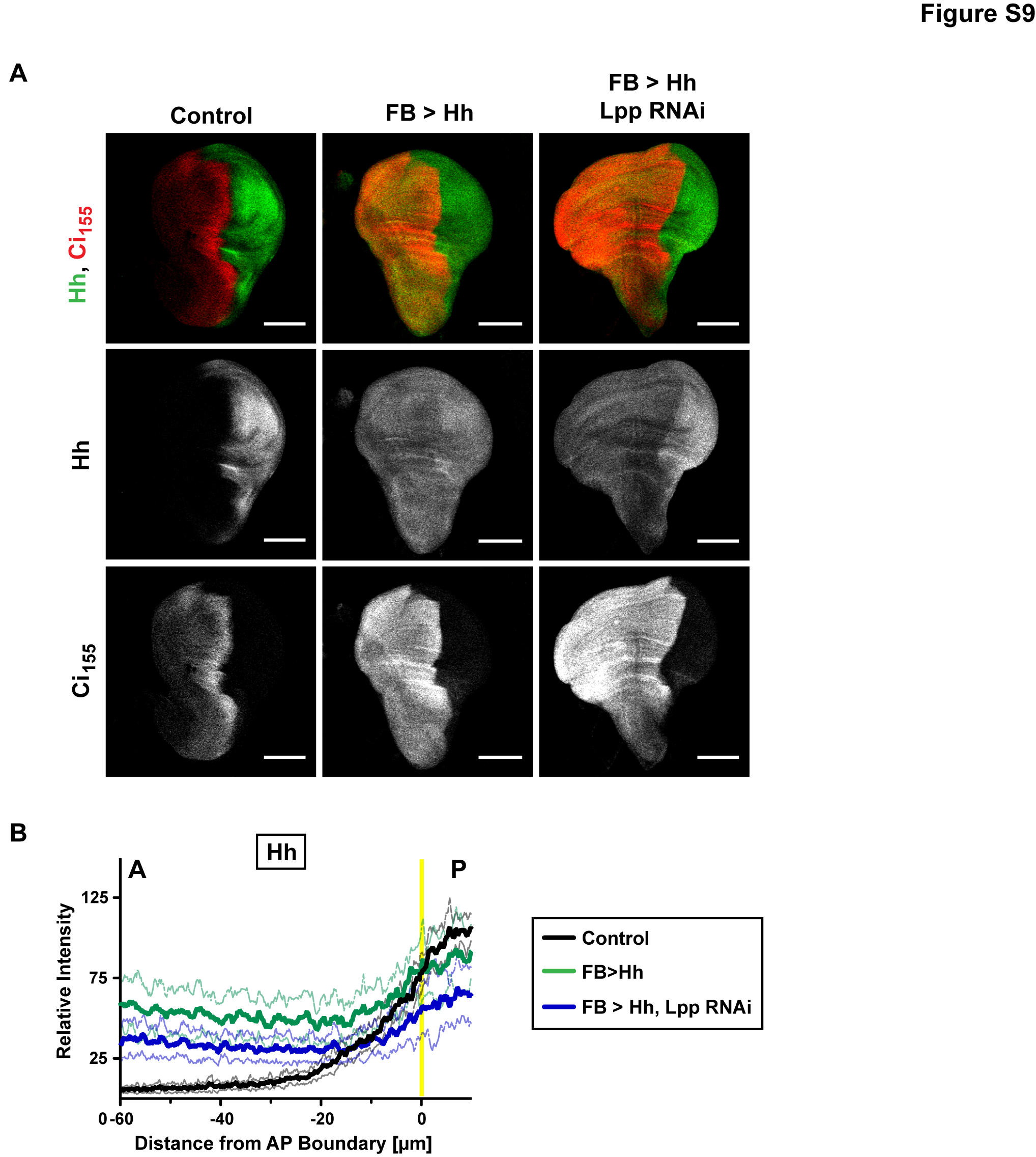

Supplement: Figure S9 — Signaling properties of Hh-N*. (A) Immunofluorescence of wing discs from larvae secreting Hh or Hh-N* from the fat body, stained for Hh and Ci155. Hh-N* was generated by expressing Hh in the fat body of Lpp RNAi animals. Scale bar = 100 µm. (B) Quantification of Hh staining of wing discs shown in (A). Yellow lines indicate the anterior/posterior compartment boundary. Translucent lines indicate ±SD (n = 10). (TIF) [file pbio.1001505.s009.tif]

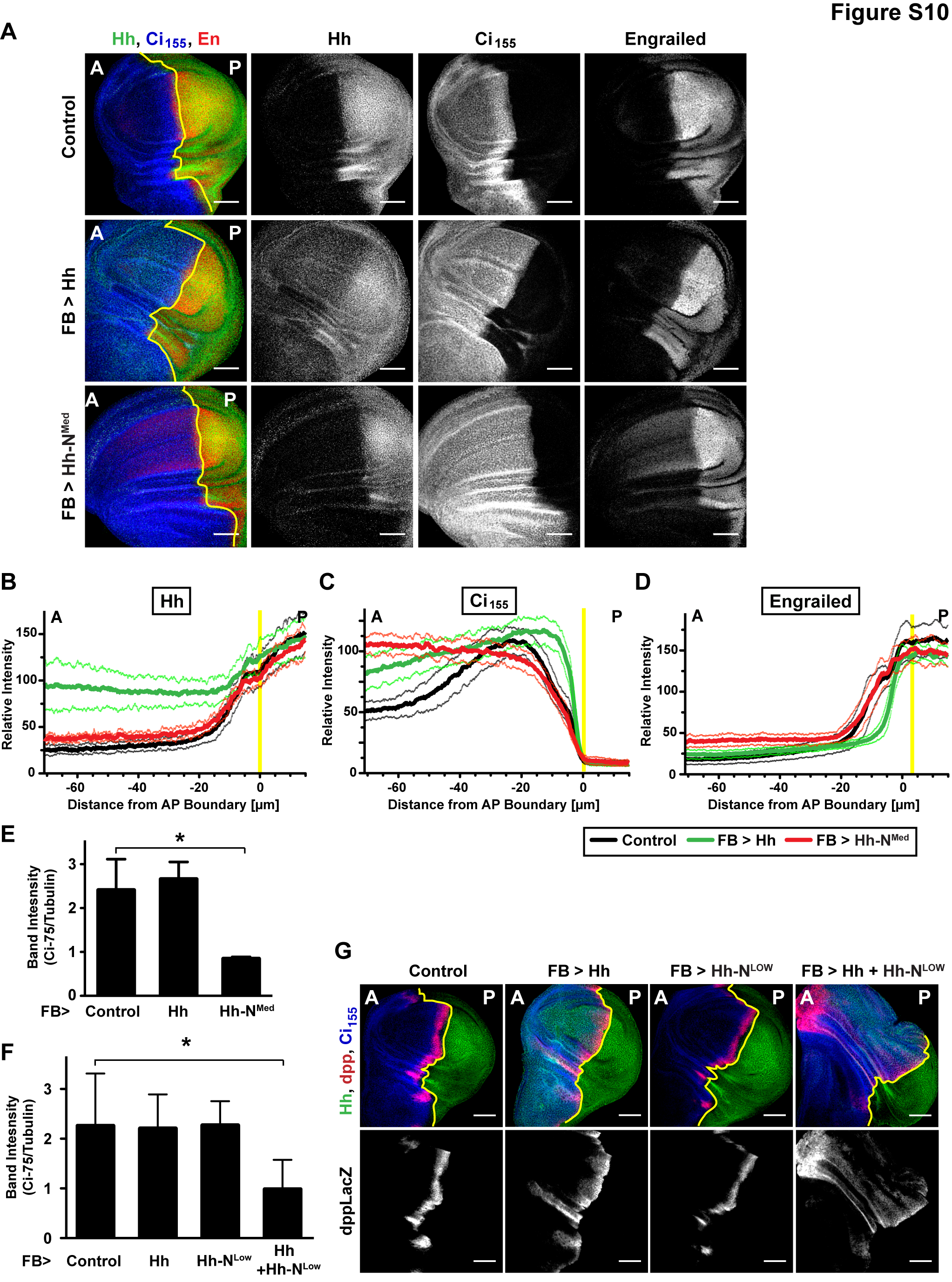

Supplement: Figure S10 — Signaling properties of Hh-N. (A) Immunofluorescence of wing discs from larvae secreting Hh or Hh-NMed from the fat body, stained for Hh, Ci155, and Engrailed. Scale bar = 50 µm. (B–D) Quantification of (B) Hh, (C) Ci155, and (D) Engrailed staining of wing discs shown in (A). Translucent lines indicate ±SD (n = 12). (E) Quantification of Ci75 levels in wing discs from larvae expressing Hh or Hh-NMed in the fat body. Band intensity of Western blots was quantified and normalized to α-tubulin. Error bars indicate ±SD (n = 3); *p<0.05. (F) Quantification of Ci75 levels in wing discs from larvae expressing Hh and Hh-NLow, alone or in combination, in the fat body. Band intensity of Western blots was quantified and normalized to α-tubulin. Error bars indicate ±SD (n = 5); *p<0.05. (G) Immunofluorescence of wing discs from larvae secreting Hh and Hh-NLow, alone or in combination, from the fat body, stained for Hh, Ci155, and dppLacZ. Scale bar = 50 µm. (TIF) [file pbio.1001505.s010.tif]

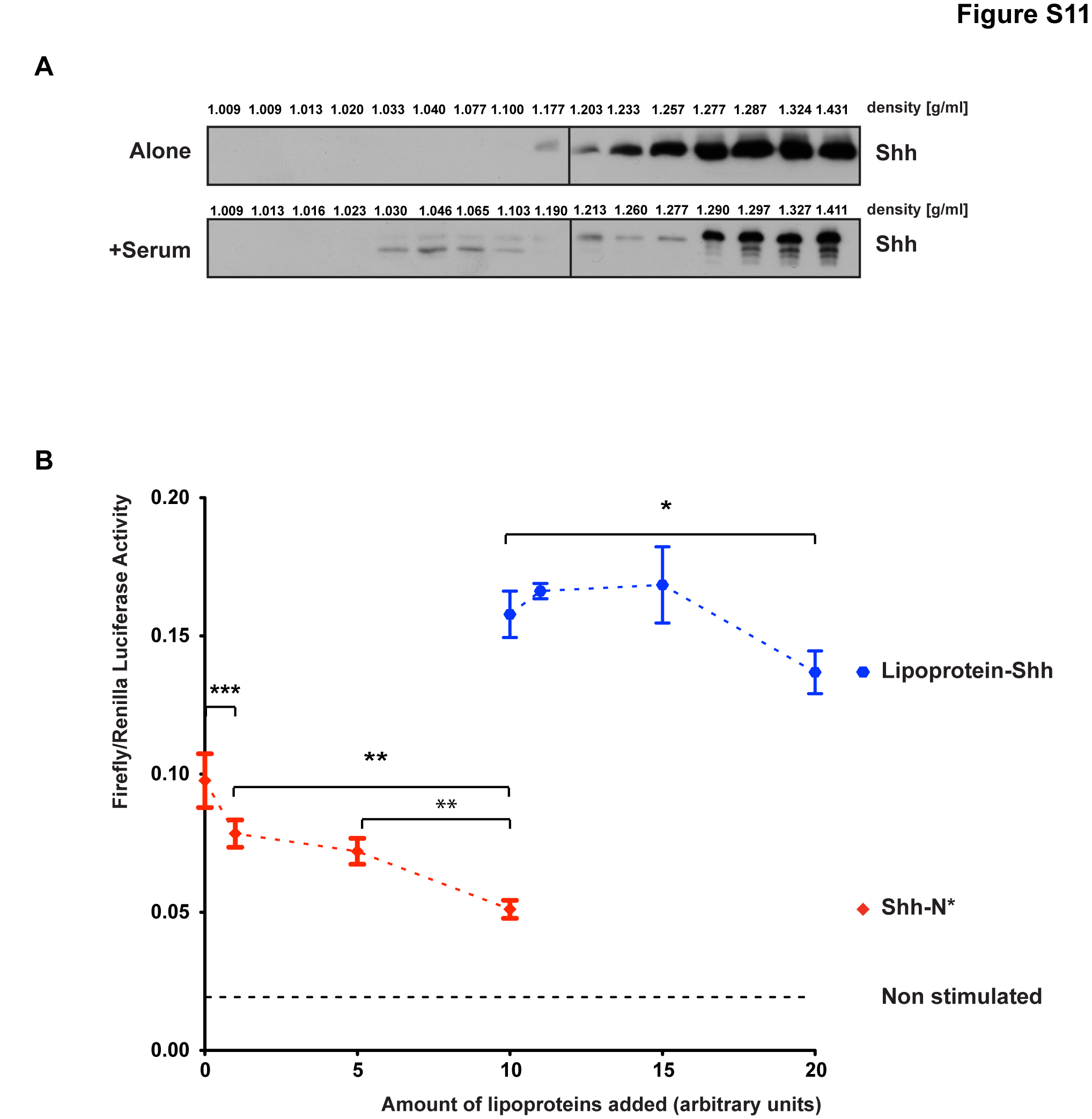

Supplement: Figure S11 — Lipoproteins repress the signaling activity of Shh-N*. (A) Shh-N* isolated from HeLa cells grown in serum-free medium was incubated in PBS containing 1% FBS at 37°C with constant shaking and subsequently fractionated by density gradient centrifugation. Shh-N* that was not exposed to serum served as a control. After incubation with serum, the vast majority of Shh-N* was still present in fractions of the highest density, suggesting that it failed to associate with lipoproteins. (B) Concentration-dependent repression of signaling activity of Shh-N* by lipoproteins in Shh-LIGHT2 cells. The concentration of lipoprotein-associated Shh or Shh-N* was kept constant, and only the amount of lipoproteins increased. Note that the starting amount of lipoproteins in the case of lipoprotein-associated Shh already corresponds to 10 arbitrary units. Error bars indicate ±SD (n = 3; *p<0.05, **p<0.005, ***p<0.0005). Shown is one representative experiment out of three. (TIF) [file pbio.1001505.s011.tif]

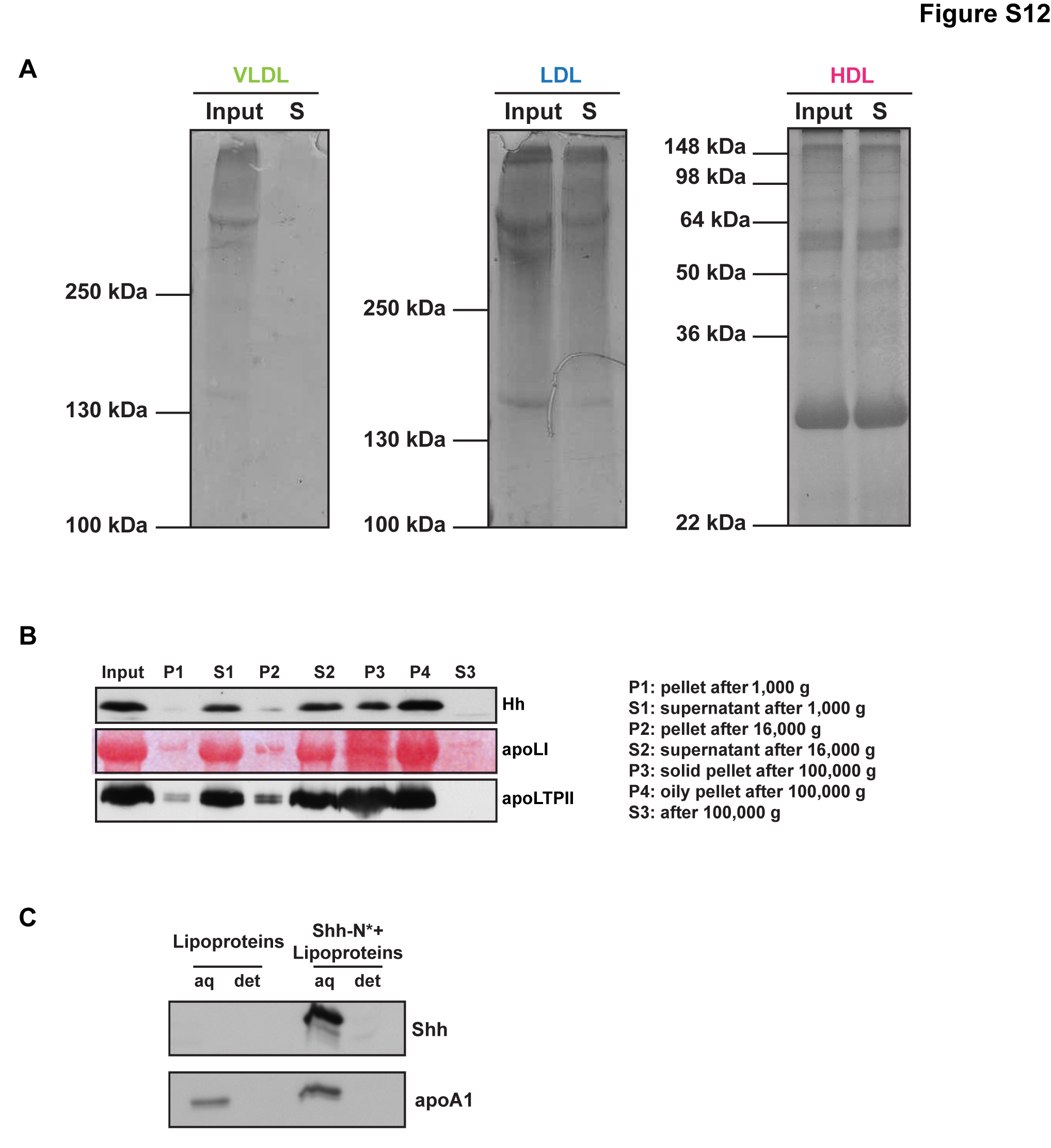

Supplement: Figure S12 — Ultracentrifuge sedimentation and Triton X-114 phase separation behavior of lipoproteins and Hh proteins. (A) Different human lipoprotein classes isolated from human serum were centrifuged at 100,000 g for 2 h. Equal volumes of input and 100,000 g supernatants (S) were separated by gel electrophoresis and visualized by Coomassie staining. Note that centrifugation at 100,000 g completely pellets VLDL and partially pellets LDL particles. HDL levels in 100,000 g supernatants are not significantly affected compared to input samples. (B) Drosophila larval hemolymph was centrifuged at the indicated speeds; resulting pellets and supernatants were analyzed by WB. The Lpp scaffolding protein apoLI was visualized by Ponceau S staining of the nitrocellulose membrane, the LTP scaffolding protein apoLTPII by immunodetection. Note that all detectable LTP and a large fraction of the smaller Lpp are pelleted by centrifugation at 100,000 g. (C) Lipoproteins isolated from FBS were subjected to Triton X-114 phase separation, alone or after mixing with Shh-N*. Note that both the HDL scaffolding apolipoprotein apoA1 and Shh-N* partition into the aqueous phase. Similarly, Shh-N* partitions into the aqueous phase in the absence of lipoproteins (see Figure 3G). (TIF) [file pbio.1001505.s012.tif]
